# Detection of grey zones in inter-rater agreement studies

**DOI:** 10.1186/s12874-022-01759-7

**Published:** 2023-01-05

**Authors:** Haydar Demirhan, Ayfer Ezgi Yilmaz

**Affiliations:** 1 Mathematical Sciences Discipline, School of Science, RMIT University, Melbourne, 3000 Victoria Australia; 2 Department of Statistics, Hacettepe University, Beytepe, Ankara, 06000 Turkey

**Keywords:** Cohen’s Kappa, Gray zone, Inter-rater reliability, Ordinal levels, Transition zone, Weighted kappa

## Abstract

**Background:**

In inter-rater agreement studies, the assessment behaviour of raters can be influenced by their experience, training levels, the degree of willingness to take risks, and the availability of clear guidelines for the assessment. When the assessment behaviour of raters differentiates for some levels of an ordinal classification, a grey zone occurs between the corresponding adjacent cells to these levels around the main diagonal of the table. A grey zone introduces a negative bias to the estimate of the agreement level between the raters. In that sense, it is crucial to detect the existence of a grey zone in an agreement table.

**Methods:**

In this study, a framework composed of a metric and the corresponding threshold is developed to identify grey zones in an agreement table. The symmetry model and Cohen’s kappa are used to define the metric, and the threshold is based on a nonlinear regression model. A numerical study is conducted to assess the accuracy of the developed framework. Real data examples are provided to illustrate the use of the metric and the impact of identifying a grey zone.

**Results:**

The sensitivity and specificity of the proposed framework are shown to be very high under moderate, substantial, and near-perfect agreement levels for $3\times 3$ and $4 \times 4$ tables and sample sizes greater than or equal to 100 and 50, respectively. Real data examples demonstrate that when a grey zone is detected in the table, it is possible to report a notably higher level of agreement in the studies.

**Conclusions:**

The accuracy of the proposed framework is sufficiently high; hence, it provides practitioners with a precise way to detect the grey zones in agreement tables.

**Supplementary Information:**

The online version contains supplementary material available at 10.1186/s12874-022-01759-7.

## Background

The level of agreement between two or more raters is considered a crucial indicator for assessing the validity of measurements that can stem from treatment responses, diagnostic scans and tests, the use of new therapeutic or diagnostic technologies, or any other quantitative procedure. Agreement studies are conducted for either discriminating between the patients (reliability) or evaluating the effects or changes through repeated measurements (agreement) [1]. In this context, the level of the agreement indicates the degree of similarity or dissimilarity between diagnoses, scores, or judgments of raters [1, 2]. Diagnostic imaging is one of the important areas where a gold standard decision criterion is not available, and agreement studies are employed to evaluate the objectivity of imaging results [3]. In pathology, the development of grading schemes is informed by the agreement studies [4]. Pathologists’ reproducibility in grading tumors is evaluated using the level of agreement between raters [5]. In cardiology, inter-rater agreement studies are employed in distinguishing type 1 and type 2 myocardial infarction due to the lack of solid clinical criteria for this classification [6, 7]. In clinical psychology, agreement studies are used to evaluate the replicability of diagnostic distinctions obtained with a diagnostic interview for mental disorders [8, 9]. In forensic medicine, the degree of agreement between two raters is utilized in the assessment of the credibility of physical torture allegations [10]. Agreement studies provide a wide variety of medical fields with essential information for critical decision-making and evaluation. Therefore, it is crucial to estimate the level of agreement between the rates with substantial accuracy.

While conducting an agreement study, one of the main concerns is the measurement scale of the outcome, which can be nominal, ordinal, interval or ratio scale. Gwet [11] outlines the selection of agreement measures to be used for different scales. In this study, we focus on cross tables (agreement tables) composed of ratings of two raters into ordinal levels. When the outcome is ordinal, the raters classify subjects into categories considering their hierarchy. Due to the impact of the hierarchy, the weighted agreement coefficients are used for ordinal outcomes [11]. The impact of different table structures on five weighted agreement coefficients is explored by Tran et al. [12]. Warrens [13–15] present theoretical and numerical results on the relationship between different weighted agreement coefficients and their usage in agreement studies. The accuracy of the weighted agreement coefficients is affected by the characteristics of the agreement table, such as unbalancedness of the counts’ distribution across the cells, the degree of true agreement, or other rater-related issues such as the existence of a grey zone [12, 16].

The assessment of raters is prone to biases due to some external factors which can be related to their personal background. The rater (examiner or observer) bias increases the variation in the raters’ assessment. This issue is explored by Schleicher et al. [17] in clinical exams in medical schools. The existence of substantial variation due to the lack of clear procedures leading to the rater bias is reported in the literature [4, 18]. Personal characteristics of the raters, such as level of expertise, their previous training, or willingness to take risks, are also sources of variation for rater bias. For example, in grading a tumor into “Normal,” “Benign disease,” “Suspected cancer,” and “Cancer” categories, one of the raters may take a cautious approach and tend to grade toward “Suspected cancer” and “Cancer” categories not to take risk while the other rater rates lower towards “Benign disease” and “Suspected cancer” categories. This difference in the willingness of raters to take risks can create a rater bias leading to grey zones such as discussed by Tran et al. [16] using data from Boyd et al. [19]. Zbären [20] reports increased accuracy in the assessment of frozen section samples with increasing experience of pathologists. In histologic grading, the distribution of grades varies up to 27% between the studies [21]. Although some portion of this variation is attributed to the patient characteristics, inter- and intra-rater variations have an extensive share in the variation. Strategies such as the use of e-learning modules to mitigate the variation caused by rater variation in grading lesions are proposed to mitigate the impact of such grey zones [22].

Since the grey zone is a concept that occurs for ordinal outcomes, we focus solely on the agreement for ordinal outcomes. The issue of having a grey zone in an agreement table is studied by Tran et al. [16, 23]. We get misleading estimates of the level of agreement when there is a grey zone in the agreement table, especially if the level of the true agreement is not high and the number of classes is not large [16]. When the sample size increases, the negative impact of a grey zone on agreement coefficients’ accuracy increases [16]. Tran et al. [23] propose a Bayesian approach for accurate estimation of the agreement level between raters when there is a grey zone in the table for ordinal tables with and without order restrictions on the levels of the classification. While the existence of grey zones in agreement tables and their negative effects are considered in the literature, the question of how we can decide whether there is any grey zone in an agreement table remains unanswered.

### Motivating example

In a study on the assessment of torture allegations, 202 cases are assessed for the consistency between the history of ill-treatment, the symptoms and the physical and psychological indications [10]. In a semi-quantitative assessment, two raters independently assessed the level of details in describing physical symptoms related to ill-treatment. The ordinal levels of the assessment that constitute a $4\times 4$ agreement table were “0” for “descriptions with no relevant information about physical abuse,” “1” for “descriptions with few details about physical abuse and symptoms,” “3” for “very detailed descriptions,” and “2” for “descriptions between 1 and 3.” The resulting agreement table is given by Petersen and Morentin [10] as in Table 1 (only a relevant section of this agreement table is presented here). Full details of the assessment, including the marginals of the table and proportions of agreement, are given by Petersen and Morentin [10].

**Table 1:** The agreement table for the level of details in the description of physical symptoms [10]

	Rater II			
Rater I	0	1	2	3
**0**	36	0	0	0
**1**	7	57	11	0
**2**	0	**23**	34	4
**3**	0	1	**19**	10

Boldfaced cells show possible locations of grey zones

For Table 1, linearly weighted Cohen’s kappa coefficient is 0.674, which indicates a good or substantial level of agreement ([24], see Table 5 therein). In this agreement table, Rater I tends to rate one level higher than Rater II for the mid-range of the scale 0-3. While Rater II considers 23 cases as describing a few details about physical abuse and symptoms, Rater I conceives that the same cases describe more details. Consistently, Rater I thinks 19 cases provided very detailed descriptions, while Rater II does not find those descriptions very detailed. Only in one cell of the agreement table, Rater II perceives more details (in 11 cases) about physical abuse and symptoms than Rater I. In this example, the perception of Rater II for the details of physical abuse and symptoms differs from that of Rater I, who shows more sensitivity to the details of physical abuse and symptoms and accepts the descriptions as *details* easier than Rater II. Overall, Rater I tends to rate one level higher than Rater II. This difference in raters’ perception creates two grey zones in this agreement table, the first one is between levels 1 and 2, and the second one is between levels 2 and 3. Petersen and Morentin [10] mention the existence of a grey zone between two levels of scoring by neither formally referring to any criteria or defining the grey zone. Identification of grey zones in such a critical area of assessment is extremely important when an assessment of allegations of torture and ill-treatment, based on the Istanbul Protocol, is required by the juridical system.

Tran et al. [16] suggest using Gwet’s AC2 or Brennan-Prediger’s S coefficients with quadratic weights when there is a grey zone in the agreement table. Cohen’s kappa, Gwet’s AC2, and Brennan-Prediger’s S coefficients are calculated using linear and quadratic weights in Table 2. Gwet’s AC2 and Brennan-Prediger’s S coefficients show a higher level of agreement with both sets of weights. Thus, if we can detect the existence of a grey zone in this agreement table in a quantitative way, we gather evidence to rely on Gwet’s AC2 and Brennan-Prediger’s S coefficients; and hence, can report a higher level of agreement that can be qualified as the very good magnitude of agreement instead of good.

**Table 2:** Weighted agreement coefficients for the agreement table given in Table 1

	Agreement coefficient		
Weight	Cohen’s kappa	Gwet’s AC2	Brennan-Prediger’s S
**Linear**	0.674	0.759	0.739
**Quadratic**	0.799	0.884	0.865

It is possible to extend such examples of agreement tables reported in the literature where a lower level of agreement is reported due to the impact of a grey zone without noticing its presence [25, 26]. In this sense, a method to detect the existence of grey zones in agreement tables helps the practitioners judge the reliability of the magnitude of agreement revealed by straightforwardly using Cohen’s kappa coefficient and leads them to use robust coefficients against the grey zones. In this study, we develop a framework to assess the existence of grey zone in ordinal agreement tables. The proposed framework is easy to implement for practitioners. It detects grey zones with high accuracy. It also has a low error rate for false detection of grey zones when there is no grey zone present in the table. We demonstrate by real data applications that a practitioner can report a higher degree of agreement between the raters with confidence when the existence of a grey zone is ascertained by the proposed framework. This leads to a better judgment of the objectivity of results or reproducibility of assessors in grading samples.

The main contribution of this study is to introduce a straightforwardly applicable framework for assessing the existence of a grey zone in an ordinal agreement table. The required software codes for calculation are presented in this article (see Supplementary Material). In this framework, a metric and a threshold are developed to detect grey zones. The sensitivity, specificity, false positive, and false negative rates of the developed metric are investigated by a numerical study. Real data applications are presented to demonstrate the usefulness of the proposed framework in practice. Using this approach, the practitioners will be able to assess the existence of a grey zone in their agreement table of interest and report more accurate agreement levels by using robust agreement coefficients against the grey zones.

## Methods

### Agreement table and grey zone

When two raters assign *n* objects into *R* classes, we get an agreement table as shown in Table 3, where $n_{ij}$ denotes the number of objects that are assigned to class *i* by the first rater and assigned to class *j* by the second rater with $i,j=1,2,\dots ,R$. The corresponding cell probability is $p_{ij}=n_{ij}/n$. The row and column totals are shown as row and column margins, respectively. Marginal row and column probabilities are $p_{i.}=n_{i.}/n$ and $p_{.j}=n_{.j}/n$, respectively. In this study, we assume that the raters are assessing ordinal levels.

**Table 3:** The agreement table for two raters

		Rater II				Row
		1	2	$\dots$	R	Margin
Rater I	1	$n_{11}$	$n_{12}$	$\dots$	$n_{1R}$	$n_{1.}$
	2	$n_{21}$	$n_{22}$	$\dots$	$n_{2R}$	$n_{2.}$
	$\vdots$	$\vdots$	$\vdots$	$\ddots$	$\vdots$	$\vdots$
	R	$n_{R1}$	$n_{R2}$	$\dots$	$n_{RR}$	$n_{R.}$
	Column Margin	$n_{.1}$	$n_{.2}$	$\dots$	$n_{.R}$	*n*

When there is complete agreement between the raters, $n_{ij}=0$ for $i\ne j$. Any deviance from this is considered as disagreement. The general form for weighted agreement coefficients ($AC_w$) for ordinal tables is defined in Eq. (1):

1 $$\begin{aligned} AC_w=\frac{P_{o}-P_{e}}{1-P_{e}}, \quad P_{o}=\sum \limits _{i,j=1}^{R}w_{ij}p_{ij}, \end{aligned}$$

where $P_o$ is the observed agreement, $P_e$ is the proportion agreement expected by chance, and $w_{ij}$ shows the weight assigned to cell (*i*, *j*) of the agreement table. There are many different versions of weighted agreement coefficients and the weights used along with them to define weighted agreement coefficients (see Tran et al. [16] for details). In this study, since each weighted agreement coefficient has its advantages and disadvantages under the existence of grey zones, we straightforwardly use the Kappa coefficient with $P_{e}$ defined in Eq. (2) and $w_{ij}=1$:

2 $$\begin{aligned} P_{e}=\sum \limits _{i,j=1}^{R}w_{ij}p_{i.}p_{.j}. \end{aligned}$$

An alternative way of assessing the level of agreement between raters is to use the *ordinal quasi-symmetry model* [27], represented by Eq. (3):

3 $$\begin{aligned} \log (p_{ij}/p_{ji})=\beta (u_{i}-u_{j}) \quad \text {for all } i \text { and } j, \end{aligned}$$

where $u_{1}\le u_{2} \le \cdots \le u_{R}$ are ordered scores assigned to the levels of the assessment scale for both row and columns of the agreement table. For this model, as the value of $|\beta |$ increases, the difference between $p_{ij}$ and $p_{ji}$ and between the marginal distributions of the raters become greater. When $\beta =0$, we have the *symmetry model* [27], which implies that the lower and upper triangles of the agreement table perfectly match, and there is a perfect fit on the main diagonal. The maximum likelihood fit of the symmetry model raises the expected cell frequencies in Eq. (4):

4 $$\begin{aligned} \hat{\mu }_{ij}=(n_{ij}+n_{ji})/2, \end{aligned}$$

and the corresponding standardised residuals are

5 $$\begin{aligned} r_{ij}=(n_{ij}-\hat{\mu }_{ij})/\sqrt{\hat{\mu }_{ij}}, \end{aligned}$$

for $n_{ij}>0$ and $r_{ij}=0$ for $n_{ij}=0$. Moreover, we have $r_{ij}=-r_{ji}$ and $r_{ij}=0$ for $i=j$.

A *grey zone* is defined as a human-contrived disagreement between two assessors occurring locally in adjacent categories of an agreement table due to subjective evaluation of raters [23]. Lack of uniform guidelines for classification, the level of experience of raters, low variability among the levels or other biases impacting the classification behaviour of raters are potential causes of not clearly distinguishing two adjacent categories. Therefore, for the grey zones considered in this study, the personal judgements of the assessors are influential on the existence of a grey zone rather than the characteristics of subjects related to a diagnosis. A grey zone is an attribute of the raters rather than being an attribute of the given scale. It is assumed that a grey zone occurs without human error, and there are no biases or misclassifications in the agreement table causing the existence of a grey zone. The causes of different grading behaviours include having different perceptions of the distance between the adjacent levels for the raters due to using different guidelines or having different experience levels. Northrup et al. [4] and van Dooijeweert et al. [18] report cases where pathologists refer to different references to grade the films leading to increased variation.

### Detection of a grey zone

We need to consider the impact of having a grey zone and how it raises simultaneously to detect a grey zone in an agreement table. The main impact of the grey zone is increased variation and uncertainty. Grey zones cause the researchers to estimate the level of agreement as lower than its actual level since inflation occurs on the off-diagonal cells of an agreement table.

When the ratings of two observers are taken as matched pairs due to the dependency created by the fact that the same subjects (diagnostic results, scans, etc.) are being rated by two raters using the same scale, we can utilise the symmetry or ordinal quasi-symmetry models for square tables to assess the existence of grey zones. The ordinal quasi-symmetry model fits the agreement table well if ratings tend to be consistently higher by one rater than the other. Since the grey zones do not occur around all the diagonal cells, the quasi symmetry model is not expected to give a satisfactory fit to detect grey zones. However, the symmetry model represents the case where there is no grey zone in the table. Therefore, if the symmetry model fits an agreement table well, it is a strong indication of not having any grey zones in the table. Following this logic, *deviations from the symmetry model for adjacent cells relative to the corresponding cells on the main diagonal* leads us to detect the existence of a grey zone in an agreement table.

The cell counts on the main diagonal stem from the agreement of two raters. For the existence of a grey zone, some cell counts should move from the main diagonal to the adjacent cell to the right (or below) of the main diagonal cell. This is a deviance from the symmetry model and penalises any agreement coefficient. Therefore, we need to consider the level of agreement along with deviations from the symmetry model to detect a grey zone. The standardised residuals of the symmetry model represent the deviations, while a kappa coefficient shows the level of agreement between the raters. There are many different forms of weighted agreement coefficients that have pros and cons depending on different formations of the agreement table and choice of weights. In fact, all the agreement coefficients will be impacted by the grey zone if it is present in the table. They all underestimate the level of agreement when there is a grey zone. Here, we only aim to represent the level of agreement instead of precisely measuring it. Since we aim to point out the difference between agreements and disagreement on the main diagonal of the table, the use of the kappa coefficient with $w_{ij}=1$ is a suitable and straightforward choice [28].

The basic element of our criterion to detect whether there is a grey zone in the agreement table or not, namely $\delta _{ij}$, is defined as the deviation from the symmetry model relative to the level of agreement measured by Cohen’s kappa coefficient as given in Eq. (6):

6 $$\begin{aligned} \delta _{ij}=r_{ij}/\kappa , \end{aligned}$$

where $r_{ij}$ is the standardised residual defined in Eq. (5) and $\kappa$ is the Cohen’s kappa coefficient. When there is a grey zone, say in the cell (*i*, *j*), the corresponding cell count, $n_{ij}$, gets inflated while $n_{ji}$ remains the same. This results in large deviance; hence, a large standardised residual, $r_{ij}$, from the symmetry model. However, the magnitude of inflation is not always due to a grey zone. It can also be related to the disagreement between the raters. Therefore, we scale the magnitude of deviance from symmetry by the level of agreement. Thus, the statistic, $\delta _{ij}$, measures the relative magnitude of deviance from the perfect agreement to the level of agreement for the cell (*i*, *j*). Then, we focus on the maximum of $\delta _{ij}$ values to detect the existence of a grey zone, and the corresponding *i* and *j* lead us to the location of the grey zone in the table. Thus, the proposed criterion to detect a grey zone is

7 $$\begin{aligned} \Delta = \max (\delta _{ij}). \end{aligned}$$

In order to give numerical insight into this approach, we focus on the agreement table given Table 1. We arbitrarily move the frequencies of the cells (shown in italic) that are potentially contributing to the grey zones to the main diagonal to create an agreement table that does not have grey zones as in Table 4. The Cohen’s kappa is calculated as $\kappa = 0.725$ and 0.545 for Tables 4 and 1, respectively. The corresponding standardised residuals for Tables 4 and 1 are shown in Table 5.

**Table 4:** The modified version of the agreement table to remove the grey zone for the level of details in the description of physical symptoms [10]

	Rater II			
Rater I	0	1	2	3
**0**	36	*0+4*=**4**	0	0
**1**	*7-4*=**3**	*57+13*=**70**	11	0
**2**	0	*23-13*=**10**	*34+14*=**48**	4
**3**	0	1	*19-14*=**5**	10

Cells with boldface and italic numbers show the modifications done to cell counts

**Table 5:** The standardized residuals of symmetry model for the agreement tables in Tables 4 and 1

No grey zone (Table 4)					With grey zone (Table 1)				
	Rater II					Rater II			
Rater I	0	1	2	3	Rater I	0	1	2	3
**0**	0	0.267	0	0	**0**	0	-1.871	0	0
**1**	-0.267	0	0.154	-0.707	**1**	1.871	0	-1.455	-0.707
**2**	0	-0.154	0	-0.236	**2**	0	1.455	0	-2.212
**3**	0	0.707	0.236	0	**3**	0	0.707	2.212	0

The magnitudes of standardized residuals are considerably higher in the table that has grey zones (Table 1) than those of the one without grey zones (Table 4). The corresponding $\delta _{ij}$ values are given in Table 6.

**Table 6:** The $\delta _{ij}$ values for Tables 4 and 1

No grey zone (Table 4)					With grey zone (Table 1)				
	Rater II					Rater II			
Rater I	0	1	2	3	Rater I	0	1	2	3
**0**	0	0.369	0	0	**0**	0	-3.433	0	0
**1**	-0.369	0	0.213	-0.975	**1**	3.433	0	-2.670	-1.297
**2**	0	-0.213	0	-0.325	**2**	0	2.670	0	-4.058
**3**	0	0.975	0.325	0	**3**	0	1.297	4.058	0

The values of the criterion $\Delta$ are 0.975 and 4.058 for Tables 4 and 1, respectively. For the assessment of torture allegations data (Table 1), we observe a very large $\Delta$ value suggesting the existence of a grey zone, as also noted by Petersen and Morentin [10]. With the arbitrarily created no-grey-zone version of the table (Table 4), we observe a very low value for $\Delta$ suggesting the absence of a grey zone in the table. These results are consistent with the logic behind the proposed criterion. However, the question we need to clarify is how large should $\Delta$ be to suggest the presence of a grey zone in the table. In order to answer this question, we develop a threshold for $\Delta$ via numerical experiments in the next section.

#### Derivation of a threshold for $\Delta$

The numerical experiments to develop a threshold for $\Delta$ of Eq. (7) involve creating random agreement tables without a grey zone. For the random generation of agreement tables, we follow the algorithm given by Tran et al. ([16], see Algorithm 1 therein) that creates bivariate normal distributed latent variables for a given Pearson correlation coefficient $(\rho )$ to set the level of not-chance-corrected true agreement between two raters.

We set $\rho = 0.45, 0.50,\dots ,0.85,0.90$ to cover true agreement levels from low to very high and consider the sample sizes of $n= 50, 100, 200, 300, 400, 500$, and 1000. Then, for each combination of $(\rho ,n)$, we generate 1000 random agreement tables (replications) that do not have any grey zone, record Cohen’s $\kappa$ and $\Delta$, and calculate minimum, maximum, and median of $\kappa$, minimum, maximum, median, and 90th and 95th percentiles of $\Delta$ over 1000 replications. The calculated values are presented in Table S1 of Supplementary file for $n=100$ and the results for all $(\rho ,n)$ pairs are tabulated in Table S1 of Supplementary file. This data generation aims to figure out the relationship between the level of agreement, sample size, $\kappa$, and $\Delta$ when there is no grey zone in the table.

**Table 7:** Descriptive statistics of $\kappa$ and $\Delta$ calculated for $n=100$ against the values of $\rho$

	$\kappa$			$\Delta$				
$\rho$	**Min**	**Med**	**Max**	**Min**	**Med**	**90th**	**95th**	**Max**
0.45	-0.002	0.213	0.442	4.564	16.053	246.335	413.388	413.388
0.50	0.008	0.231	0.444	4.585	13.777	376.726	376.726	376.726
0.55	0.053	0.263	0.497	3.998	10.750	158.032	158.032	249.900
0.60	0.068	0.292	0.525	3.792	9.013	43.027	104.885	181.672
0.65	0.082	0.331	0.591	3.702	7.416	16.878	16.878	146.471
0.70	0.142	0.371	0.621	2.852	6.363	10.605	14.711	46.144
0.75	0.157	0.418	0.635	2.288	5.438	6.938	6.938	29.757
0.80	0.253	0.471	0.771	1.456	4.690	5.238	5.298	12.260
0.85	0.332	0.532	0.771	1.385	3.878	4.630	4.630	8.992
0.90	0.410	0.607	0.831	1.593	3.203	4.349	4.349	7.542

*Min* minimum, *Max* maximum, *Med* median, *90th* 90th percentile, *95th* 95th percentile

From Table 7, the value and the range of $\Delta$ decreases as the level of agreement increases for $n=100$. As expected, there is a clear negative correlation between the level of agreement and $\Delta$. We observe the same relationship for larger sample sizes from Table S1 of Supplementary file. As the sample size gets larger, the maximum and the range of $\Delta$ decreases. Therefore, *a sensitive threshold for* $\Delta$ *needs to be a function of both the sample size and the level of agreement.*

**Fig. 1:**
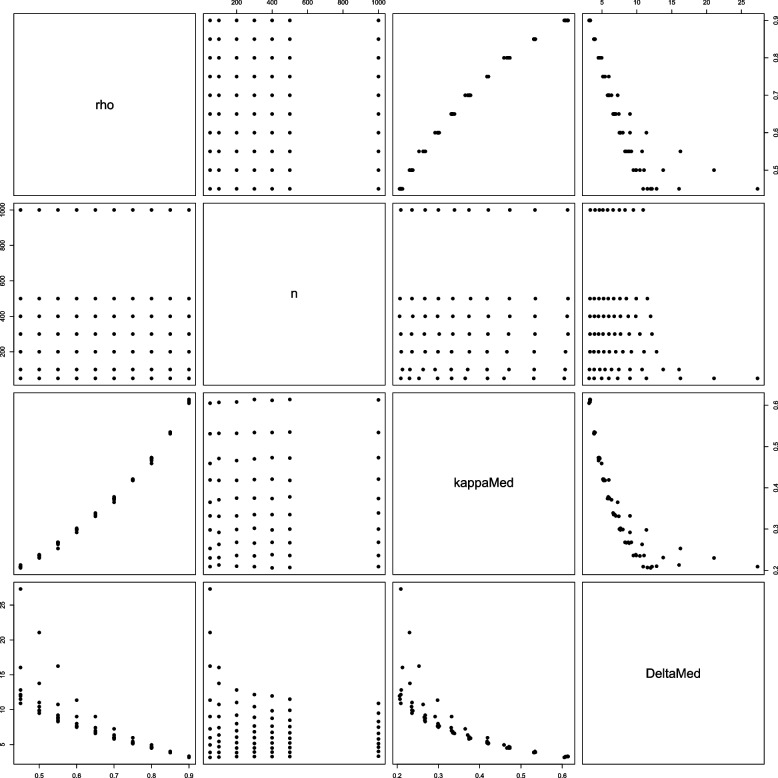
Scatter plots of the pairs of $\rho$ (rho), sample size (*n*), the median of $\kappa$ (kappaMed), and median of $\Delta$ (DeltaMed)

Scatter plots of the pairs of $\rho$, *n*, median of $\kappa$, and median of $\Delta$ are displayed in Fig. 1. The relationship patterns between median $\Delta$ and both $\rho$ and median $\kappa$ are very similar. There is a negative nonlinear relationship between the level of agreement and $\Delta$. The range of median $\Delta$ increases for smaller samples nonlinearly. Therefore, *a functional threshold needs to reflect these nonlinear relationship patterns.*

In order to develop a threshold that is a function of both the level of agreement and the sample size and incorporates the nonlinear relationships, we utilize the nonlinear regression technique. We build a model for the median $\Delta$ given the median $\kappa$ and the sample size. Although the mean is more representative, a small number of large-valued outlier observations can impact the value of the mean considerably, while the median stays unaffected. From Table S1 of the Supplementary Material, we observe a large range of $\Delta$ values for each $\rho$ among the values of sample size, *n*. Similarly, the range of $\Delta$ values for each *n* is considerably large among the considered $\rho$ values. This implies that the likelihood of getting outlier $\Delta$ values for a given agreement table is not negligible. Therefore, we used the median instead of the mean to build the nonlinear regression model to get robust results against the outliers.

**Fig. 2:**
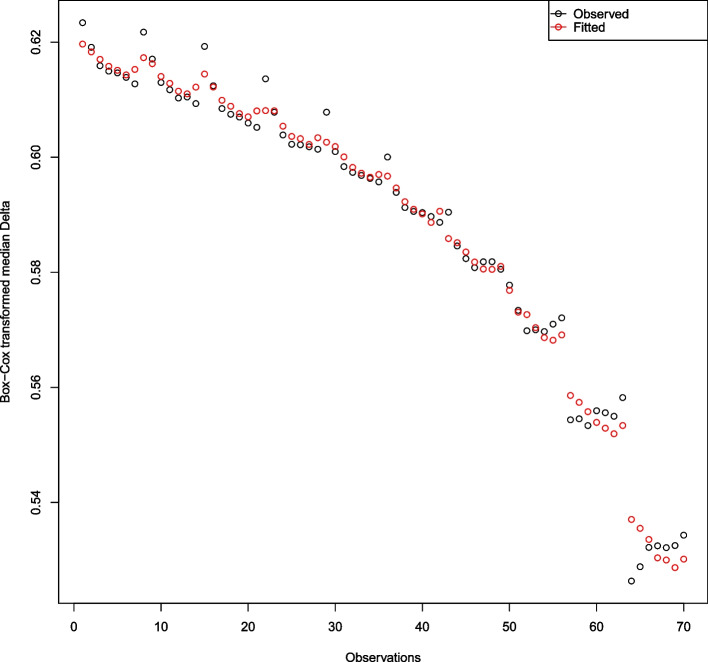
Scatter plots of the observed and fitted Box-Cox transformed median $\Delta$

In the scatter plots of both the median $\Delta$ and the median $\kappa$, and the median $\Delta$ and *n* (Fig. 1), the variation increases as the median $\Delta$ increases and the median $\kappa$ and *n* decrease. So, we apply the Box-Cox transformation [29] to stabilise this variation before moving into the modelling. The optimal value of the power parameter $\lambda$ of the Box-Cox transformation is found as -1.59596 by using the **boxcox()** function from the **MASS** R package [30]. Then, we fit the model in Eq. (8) with the Box-Cox transformed $\Delta$ values, $\Delta _{BC}$.

8 $$\begin{aligned} \Delta _{BC} = \beta _{0}+\beta _{1}\kappa ^{2} + \beta _{2}n+ \beta _{3}n^{2}+\epsilon , \end{aligned}$$

where $\epsilon \sim N(0,\sigma _{\epsilon }^{2})$. This specific model form is found by optimizing the adjusted R-squared over a model space that contains the models with linear and quadratic terms of $\kappa$ and *n*. The fitted model is obtained as

9 $$\begin{aligned} \widehat{\Delta }_{BC} = 0.6319 - 0.2563\kappa ^{2} -2.087\cdot 10^{-5}n+ 1.546\cdot 10^{-8}n^{2} \end{aligned}$$

with all statistically significant coefficients at 5% level of significance ($P<0.001$ for all). For this model, the adjusted R-squared is 0.989, which implies an almost perfect fit. Figure 2 shows the observed and fitted values by Eq. (9).

In the top-left section of Fig. 2, six observations are notably underestimated by the model in Eq. (8). These observations come from the replications with a small sample size with $n=50$. For the rest of the sample sizes, we have an almost perfect fit that is also identified by the adjusted R-squared of 0.989. Thus, when we take the Box-Cox transformation back by Eq. (10),

10 $$\begin{aligned} \tau _{\Delta }= & {} (\widehat{\Delta }_{BC} \cdot \lambda + 1)^{(1/\lambda )}\nonumber \\= & {} \left[ (0.6319 - 0.2563\kappa ^{2} - 2.087\cdot 10^{-5}n+ 1.546\cdot 10^{-8}n^{2}) \times (-1.59596) + 1\right] ^{(1/-1.59596)}\nonumber \\= & {} (-0.0080 + 0.4090\kappa ^{2} + 3.331\cdot 10^{-5}n - 2.467 \cdot 10^{-8}n^{2})^{-0.6266}, \end{aligned}$$

we observe the desired threshold, $\tau _{\Delta }$, for our criterion $\Delta$. As seen in Eq. (10), $\tau _{\Delta }$ reflects the nonlinear relationship patterns between median $\Delta$ and $\kappa$ and *n*. Since $\tau _{\Delta }$ is an estimate of $\Delta$ when there is no grey zone in the agreement table and $\Delta$ tends to increase when there is a grey zone in the table, *if* $\Delta >\tau _{\Delta }$, *then it is decided that there is a grey zone exists in the agreement table. Otherwise, there is no grey zone in the table.*

Once it is decided that there is a grey zone in the table by $\Delta >\tau _{\Delta }$, it is possible to compare other $\delta _{ij}$ values with $\tau _{\Delta }$ to identify other grey zones in the table.

## Results

### Numerical experiments

We conducted an experimental study to assess the performance of the proposed metric to detect the existence of a grey zone in an agreement table. The approach in this validation effort is to i) generate an agreement table without any grey zone, ii) introduce a grey zone into the same table without effecting the level of agreement notably, and iii) record the values of $\Delta$ for each case and compare them with the corresponding threshold calculated by Eq. (10). In this way, we reveal the true-positive (sensitivity), true-negative (specificity), false-negative, and false-positive rates of the approach proposed in this study.

#### Data generation

The approach of Muthén [31] is used along with the algorithm given by Tran et al. [16] to generate agreement tables without a grey zone. Moderate, substantial, and near-perfect levels of true agreement are generated by using the correlation coefficient $\rho$. These agreement levels respectively correspond to Cohen’s kappa values around 0.63, 0.75, and 0.83. Note that it is not possible to get exact kappa values as desired in the Monte Carlo data generation environment. The kappa values lower than 0.6 and higher than 0.85 are not feasible due to the nature of grey zones. For low true agreements, the off-diagonal cells of the table get inflated by disagreement; hence, a grey zone does not occur. For perfect true agreements, the cell counts get highly concentrated on the main diagonal of the table and do not allow the formation of a grey zone. The sample size is taken as $n = 50,100,250,500$, and 1000. For each sample size, a different value of $\rho$ gives a desired value for $\kappa$. The table size is considered as $R=3$ and 4. For larger table sizes, the ordinal scale starts to approach the continuous scale; hence, it does not inform us about the pure impact of the ordinal outcome. Johnson and Creech [32] observe that when $R>4$, the bias due to categorisation of continuous measurements does not have a substantial impact in the interpretations. Considering these, including larger table sizes is not quite informative for our aim in this study. The values of $\rho$ and corresponding $\kappa$ are tabulated for each sample size in Table 8.

**Table 8:** The values of $\rho$ and corresponding $\kappa$ values for each sample size, *n*, for $3\times 3$ tables

*n*	$\boldsymbol\rho$	$\boldsymbol\kappa$	*n*	$\boldsymbol\rho$	$\boldsymbol\kappa$
50	0.960	0.639	500	0.910	0.630
	0.980	0.756		0.960	0.754
	0.986	0.817		0.984	0.838
100	0.930	0.639	1000	0.900	0.632
	0.965	0.744		0.960	0.767
	0.985	0.835		0.980	0.832
250	0.925	0.633			
	0.963	0.753			
	0.977	0.838			

In order to inject a grey zone into an agreement table, the search approach of Tran et al. [16] is utilized on the cell probabilities for each combination of $\rho$ and *n*. We searched for the set of cell probabilities that produces a $\kappa$ value that is almost equal to that of the corresponding table without a grey zone. This way, we make sure that the generated tables with and without a grey zone have the same level of agreement for comparability.

For each table size, we consider the position of the generated grey zone. For $R=3$, the grey zone is created at cells (1, 2), (2, 1), (2, 3), and (3, 2), and for $R=4$, it is created at cells (1, 2), (2, 1), (2, 3), (3, 2), (3, 4), and (4, 3). In total, we consider 150 different scenarios composed of $\rho , n, R$ and the location of the grey zone. For each scenario, 10,000 random agreement tables with and without a grey zone are generated.

#### Accuracy of $\Delta$

We focus on sensitivity, specificity and Mathew’s correlation coefficient (MCC) to describe the accuracy of the proposed criterion. While sensitivity and specificity reflect true-positive and true-negative classifications about having a grey zone in the table, MCC considers false-positive and false-negative decisions along with true-positive and true-negative classifications. There are other performance measures such as precision and F1 score. However, since we create 10,000 tables without a grey zone and 10,000 tables with a grey zone, sensitivity, recall, and F1 score are all equal to each other. Suppose *TP*, *TN*, *FP*, and *FN* respectively show the number of true-positive, true-negative, false-positive, and false-negative decisions on the existence of a grey zone in the generated tables. Then, sensitivity, specificity, and the Mathew’s correlation coefficient [33] are calculated as in Eqs. (11) and (12):

11 $$\begin{aligned} \text {Sensitivity} = \frac{TP}{10,000}, \quad \text {Specificity}= \frac{TN}{10,000}, \quad \text {and} \end{aligned}$$

12 $$\begin{aligned} \text {MCC} = \frac{(TP\times TN)-(FP\times FN) }{[(TP+FP)(TP+FN)(TN+FP)(TN+FN)]^{0.5}}. \end{aligned}$$

**Fig. 3:**
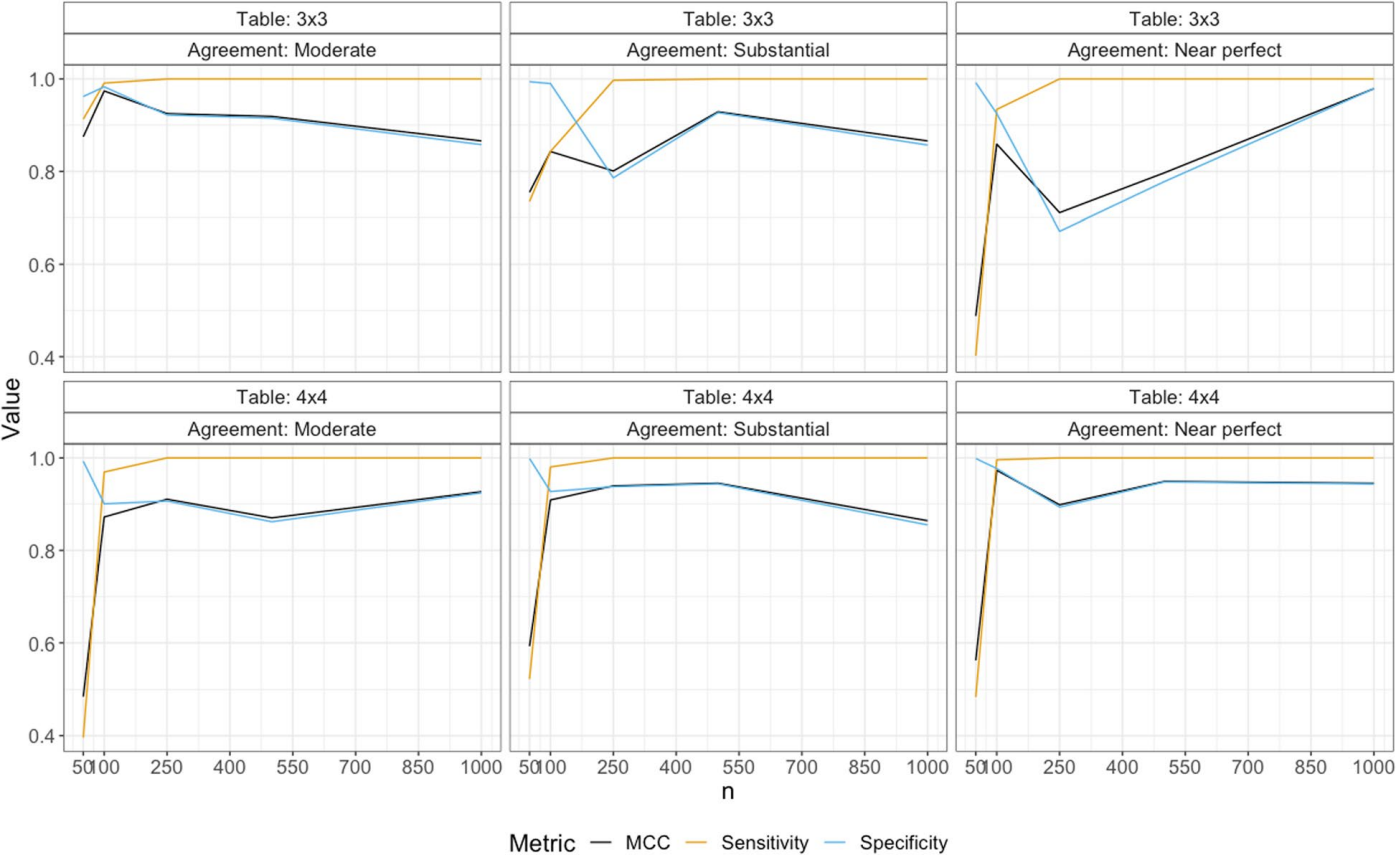
Accuracy metrics of $\Delta$ for $3 \times 3$ and $4 \times 4$ agreement tables under different true agreement levels

The proposed criterion, $\Delta$ and the threshold, $\tau _{\Delta }$, are computed for each generated table. Then, we create a classification table composed of the true and estimated status of a grey zone in the table over 10,000 replications and compute the accuracy measures in Eqs. (11) and (12). The results when the grey zone is in cell (1, 2) of the table for $R=3$ and 4 are given Table 9. MCC, sensitivity, and specificity results are plotted for low, moderate, and high agreement under $3 \times 3$ and $4 \times 4$ table settings in Fig. 3. The results for all scenarios, the cell probabilities used to inject the grey zone and the corresponding Cohen’s kappa after introducing a grey zone in the table for $3 \times 3$ and $4 \times 4$ agreement tables are given in Tables S2 and S3 of Supplementary file, respectively.

**Table 9:** Sample size, $\rho$, true $\kappa$, *TP*, *TN*, *FP*, and *FN*, sensitivity, specificity, and MCC when the grey zone is in cell (1, 2) of $3\times 3$ and $4\times 4$ agreement tables

R	Case	*n*	$\rho$	True $\kappa$	TP	FP	FN	TN	Sens	Spec	MCC
3	**GZ at cell (1,2)**	50	0.960	0.639	9126	874	385	9615	0.913	0.962	0.875
			0.980	0.756	7352	2648	58	9942	0.735	0.994	0.755
			0.986	0.817	4017	5983	80	9920	0.402	0.992	0.488
		100	0.930	0.639	9906	94	168	9832	0.991	0.983	0.974
			0.965	0.744	8432	1568	98	9902	0.843	0.990	0.843
			0.985	0.835	9338	662	749	9251	0.934	0.925	0.859
		250	0.925	0.633	10000	0	777	9223	1.000	0.922	0.925
			0.963	0.753	9973	27	2141	7859	0.997	0.786	0.801
			0.977	0.838	10000	0	3288	6712	1.000	0.671	0.711
		500	0.910	0.630	10000	0	846	9154	1.000	0.915	0.919
			0.960	0.754	10000	0	733	9267	1.000	0.927	0.929
			0.984	0.838	10000	0	2225	7775	1.000	0.778	0.797
		1000	0.900	0.632	10000	0	1424	8576	1.000	0.858	0.866
			0.960	0.767	10000	0	1430	8570	1.000	0.857	0.866
			0.980	0.832	10000	0	212	9788	1.000	0.979	0.979
4	**at cell (1,2)**	50	0.911	0.624	3958	6042	72	9928	0.396	0.993	0.484
			0.969	0.731	5224	4776	12	9988	0.522	0.999	0.593
			0.982	0.839	4839	5161	13	9987	0.484	0.999	0.563
		100	0.935	0.612	9694	306	993	9007	0.969	0.901	0.872
			0.982	0.746	9804	196	727	9273	0.980	0.927	0.909
			0.992	0.840	9959	41	229	9771	0.996	0.977	0.973
		250	0.945	0.616	10000	0	933	9067	1.000	0.907	0.911
			0.975	0.755	10000	0	623	9377	1.000	0.938	0.940
			0.987	0.824	10000	0	1065	8935	1.000	0.894	0.899
		500	0.945	0.613	10000	0	1381	8619	1.000	0.862	0.870
			0.977	0.747	10000	0	562	9438	1.000	0.944	0.945
			0.987	0.824	10000	0	522	9478	1.000	0.948	0.949
		1000	0.940	0.617	10000	0	757	9243	1.000	0.924	0.927
			0.975	0.740	10000	0	1450	8550	1.000	0.855	0.864
			0.985	0.828	10000	0	563	9437	1.000	0.944	0.945

TP: GZ+ TGZ+; FP: GZ+ TGZ-; NF: GZ- TGZ+; TN: GZ- TGZ-; TGZ+: There is a grey zone in the table; TGZ-: There is no grey zone in the table; GZ+: A grey zone is identified; in the table; GZ-: No grey zone is identified in the table; Sens: Sensitivity; Spec: Specificity; MCC: Mathew’s correlation coefficient

For small sample sizes and near-perfect level of true agreement, it is highly challenging to detect the existence of a grey zone since the cell counts moving to off-diagonal cells as the result of a grey zone are not large enough to be separated from disagreement easily. Therefore, the sensitivity of $\Delta$, namely the accuracy of detecting the existence of a grey zone correctly, is not as high as desired for $n=50$ in $3 \times 3$ tables. In $4 \times 4$ tables, it is low for $n=50$, and all true agreement levels since the sample size of 50 are distributed across 16 cells instead of 9, making it harder to detect the movement of counts. However, the sensitivity of $\Delta$ rapidly increases over 0.9 when $n\ge 100$ for both table sizes; hence, $\Delta$’s ability to detect a grey zone is very high for $n\ge 100$ for both table structures and all levels of true agreement. The same inferences follow for MCC as well. The specificity of $\Delta$, namely the accuracy of concluding the absence of a grey zone correctly, is very high for small samples and slightly reduces to around 0.9 for higher sample sizes for all true agreement levels under $4 \times 4$ table size and a moderate level of true agreement under $3 \times 3$ tables. There is a drop in specificity of $\Delta$ for moderate sample sizes under $3 \times 3$ tables and high true agreement. The reason for this is having a near-perfect agreement. When the agreement is near-perfect, if the sample size is not large, there are not many cell counts move off the diagonal to create a notable grey zone that makes it harder to detect for $\Delta$. Having near-perfect agreement and a low sample size are two extreme ends of the conditions where a grey zone can occur. From the rates of a false-positive decision in Table 9, there is almost no case where the proposed framework indicates the existence of a grey zone when there is no grey zone in the table for moderate and large sample sizes $(n\ge 250)$. However, there is an acceptable level of false negative decisions where the framework indicates that there is no grey zone in the table while a grey zone is present.

From Tables S2 and S3 of Supplementary file, we draw similar inferences for the accuracy of $\Delta$ when the location of the grey zone moves from the cell (1,2) to other possible cells. Therefore, the location of a grey zone in the agreement table does not have an impact on the accuracy of $\Delta$. Overall, the accuracy of $\Delta$ along with the threshold $\tau _{\Delta }$ in detecting the absence and presence of a grey zone is substantially high for sample sizes between 100 and 1,000 under all considered table sizes and the levels of agreement. This makes the proposed framework for detecting a grey zone a useful and reliable approach.

### Applications with real data

In order to demonstrate the use of the proposed framework for the detection of grey zones in practice, we focus on previously published agreement tables from studies in the medical field. R codes for the software implementation of the framework are given in the Supplementary Material, along with the calculations for the following applications.

#### Assessment of torture allegations

We revisit the agreement table given in the motivating example. The agreement table in Table 1 shows the classification resulting from two raters’ assessment of the level of details in the description of physical symptoms related to ill-treatment in $n=202$ cases [10]. Petersen and Morentin [10] mention that there is a grey zone in this table based on their conceptual assessment without using any metric.

In order to calculate $\Delta$ by Eqs. (6) and (7), we use the standardized residuals of the symmetry model given on the right-side of Table 5, the corresponding $\kappa = 0.545$, and $\delta _{ij}$ values in Table 6. From Eq. (7), we get $\Delta = 4.058$. Then, we need to calculate the threshold, $\tau _{\Delta }$ from Eq. (10) as follows:

13 $$\begin{aligned} \tau _{\Delta }= & {} (-0.0080 + 0.4090\kappa ^{2} + 3.331\cdot 10^{-5}n - 2.467 \cdot 10^{-8}n^{2})^{-0.6266}\nonumber \\= & {} (-0.0080 + 0.4090\cdot 0.545^{2} + 3.331\cdot 10^{-5}\cdot 202 \nonumber \\&- 2.467 \cdot 10^{-8}\cdot 202^{2})^{-0.6266}\nonumber \\= & {} 3.791. \end{aligned}$$

Since we have $\Delta = 4.058>3.791=\tau _{\Delta }$, it is decided that there is a grey zone in this agreement table. When we check the $\delta _{ij}$ values, $\delta _{43} = 4.058$. So, the highest magnitude grey zone is between levels 3 and 2, where Rater I tends to rate towards level 3 while Rater II tends to assign the cases to level 2 (note that the levels start from 0 in this data). Looking at Table 6, we observe that there is no other $\delta _{ij}$ greater than 3.791. There is only one other cell that has a $\delta _{ij}$ value close to $\tau _{\Delta }$, $\delta _{21}=3.433$, where Rater I classifies only 7 cases to level 1 while Rater II assigns them to level 2. This is consistent with Rater I’s assessment tendency of rating one level higher than Rater II. However, since raters’ level of agreement on level 0 is high, it does not create enough deviation to be identified as a grey zone. Overall, the grey zone identified in this agreement table is in accordance with the conclusions of Petersen and Morentin [10] about the existence of grey zones in this data. It is possible to report Gwet’s AC2 or Brennan-Prediger’s S with quadratic weights (Table 2) to conclude a higher level of the agreement due to the existence of a grey zone in this study.

#### Assessment of PI-RADS v2.1 scores

In a recent study, Wei et al. [26] focused on developing a graphical representation to predict significant prostate cancer in the transition zone based on the scores from the Prostate Imaging Reporting and Data System version 2.1 (PI-RADS v2.1). Wei et al. ([26], Table 2 therein) report the classification of $n=511$ cases into five levels of PI-RADS v2.1 scores by two radiologists. In this classification, Radiologist 1 tends to rate one level higher than Radiologist 2 for 2, 3, and 4 levels of PI-RADS v2.1. The Cohen’s kappa is $\kappa = 0.461$. To decide if there is a grey zone in this table, $\delta _{ij}, i,j=1,\dots ,5$ are calculated by Eq. (6) as in Table 10.

**Table 10:** The $\delta _{ij}$ values for PI-RADS v2.1 agreement table

	Radiologist 1				
Radiologist 2	1	2	3	4	5
**1**	0	4.625	3.579	1.534	0
**2**	-4.625	0	0.166	3.254	0
**3**	-3.579	-0.166	0	3.889	0
**4**	-1.534	-3.254	-3.889	0	-1.252
**5**	0	0	0	1.252	0

For Table 10, $\Delta = 4.625$ by Eq. (7). Then, $\tau _{\Delta }$ is calculated by Eq. (10) as follows:

14 $$\begin{aligned} \tau _{\Delta }&(-0.0080 + 0.4090\cdot 0.461^{2} + 3.331\cdot 10^{-5}\cdot 511 - 2.467 \cdot 10^{-8}\cdot 511^{2})^{-0.6266} = 4.537. \end{aligned}$$

Since we get $\Delta = 4.625 > 4.537 = \tau _{\Delta }$, it is concluded that there is a grey zone in the agreement table of two radiologists for PI-RADS v2.1 scores. From Table 10, $\delta _{12} = 4.625$; hence, the grey zone is in between levels 1 and 2 where Radiologist 1 tends to rate one level higher than Radiologist 2 for level 1 of PI-RADS v2.1 scores.

The practical implication of identifying this grey zone is related to the reported level of agreement. Wei et al. [26] report a weighted version of Cohen’s kappa as 0.648 that corresponds to linearly weighted kappa. However, Tran et al. [16] finds that Gwet’s AC2 and Brennan-Prediger’s S measures with the quadratic and linear weights are most robust against the grey zones. These weighted agreement coefficients are reported in Table 11 for PI-RADS v2.1 scores data.

**Table 11:** Weighted agreement coefficients for assessment of two radiologists for PI-RADS v2.1 scores

	Agreement coefficient		
Weight	Cohen’s kappa	Gwet’s AC2	Brennan-Prediger’s S
**Linear**	0.651	0.793	0.747
**Quadratic**	0.805	0.916	0.879

According to Fleis et al. [34] interpretation of the kappa coefficient, a kappa value of 0.651 corresponds to a “Fair to Good.” However, all other kappa values in Table 11 indicate one level higher, “Very Good,” agreement between two radiologists. Therefore, detecting the grey zone leads to reporting a higher level of agreement between the radiologists.

## Discussion

Grey zones arise in agreement studies in various fields from medicine to education that involves assigning the subjects to ordinal levels. When the raters or assessors tend to assign subjects into classification categories in different manners, an unbalanced structure occurs in adjacent cells around the main diagonal of the agreement table. This unbalance creates a grey zone(s) and causes reporting the level of agreement to be lower than its actual value. The negative impact of grey zones is demonstrated by Tran et al. [16, 23]. Since a grey zone is an attribute of the rating attitudes of raters, it originates from their willingness to take risks, expertise, training, or lack of uniform guidelines for the assessment. If the raters take more training after the first round of scoring or are given clearer guidelines for rating, it would be expected that they will not produce the same grey zone(s). To avoid the impact of grey zones or test such a hypothesis that more training would mitigate the occurrence of grey zones, we need an objective framework to decide if there is a grey zone in an agreement table.

This study proposes a framework that includes two statistics: a criterion and a threshold. The criterion, $\Delta$, captures the deviations from the symmetry model relative to the level of agreement. The threshold, $\tau _{\Delta }$, is a nonlinear function of the level of agreement and the sample size obtained by nonlinear regression modelling. The comparison of $\Delta$ to $\tau _{\Delta }$ provides us with a decision criterion for the identification of a grey zone in the agreement table. The accuracy of the proposed framework is tested by a numerical study through the metrics sensitivity, specificity, and Matthew’s correlation coefficient. Small, moderate, large, and very large sample sizes, moderate, substantial, and near-perfect agreement levels and $3\times 3$ and $4 \times 4$ table sizes are considered in the numerical study. Low and perfect agreement levels are not feasible settings for the existence of a grey zone since they respectively represent the cases where the raters totally disagree or agree. The tables greater than $4 \times 4$, the impact of the ordinal scale reduces and starts to approach the continuous scale [32]. Therefore, the results of our numerical study are generalizable to other cases in the grey zone concept. The proposed framework has satisfactorily high sensitivity for samples larger than 50 observations. Its specificity is very high for all sample sizes. When false-positive and false-negative rates are also considered by the use of Matthew’s correlation coefficient, we get satisfactorily high correlations for samples with larger than 50 observations.

Although the grey zone concept is defined as an attribute of the raters due to their background and assessment approach, this concept can be extended to the comparison of two diagnostic methods in the grading of diseases. Zavanone et al. [35] consider grading carotid artery stenosis using noninvasive imaging methods, Doppler ultrasound (DUS) and computed tomography angiography (CTA). They compare the classifications by DUS against CTA in grading carotid artery stenosis in 431 arteries into the levels of “Mild”, “Moderate”, “Severe”, and “Occlusion”. The expectation is to see some degree of agreement between the methods in the grading of the same arteries into the same scale. Although these imaging methods cannot have any biases, they have some differences due to the ways they work, and this can raise artificial inflation in adjacent cells around the main diagonal of the agreement table. Zavanone et al. ([35], Table 1 therein) report the agreement table of DUS and CTA in grading carotid artery stenosis. DUS tends to rate more towards “Severe” in the raw table, while CTA rates those cases as “Moderate”. For this table, $n=431$ and $\kappa = 0.674$. When we implement the proposed framework, we get $\Delta = 3.028$ and $\tau _{\Delta }=2.853$. Since $\Delta = 3.028>2.853=\tau _{\Delta }$, we conclude that there is a grey zone between the levels “Moderate” and “Severe” in the grading of DUS and CTA for carotid artery stenosis. Zavanone et al. [35] report the Cohen’s quadratic weighted kappa as 0.85, which is more robust against the grey zones [16]. However, due to the identification of the grey zone, we can rely on Gwet’s AC2 and Brennan-Prediger’s S with quadratic weights, which are 0.908 and 0.887, and report even a higher agreement between DUS and CTA.

The main limitation of this study is around the nonlinear regression model used to develop the threshold for $\Delta$. The accuracy of $\Delta$ is directly related to the goodness-of-fit of the nonlinear regression model. We obtained an adjusted R-squared of 0.989 for this model. This shows a near-perfect fit for interpolation, occurring for sample sizes between 50 and 1,000 and true kappa values of -0.002 and 0.831. Therefore, the proposed framework should be used cautiously for the samples with less than 50 or more than 1,000 observations or the agreement tables with a very high true agreement. As discussed, the likelihood of having a grey zone in these cases is extremely low.

## Conclusions

In this study, a framework is proposed to detect the existence of grey zones in an agreement table. The main conclusions from the real-data examples and the experimental study conducted with $3\times 3$ and $4 \times 4$ agreement tables under small, moderate, and large samples and moderate, substantial, and near-perfect agreement levels are summarized as follows:

- The proposed framework has a sufficiently high-level capability to detect the existence of a grey zone for tables of size greater than 50 under all the considered table sizes and true agreement levels.
- The proposed framework’s accuracy in correctly determining the absence of a grey zone is very high in all the considered scenarios of sample size, table size, and the true agreement level.
- When there is no grey zone in the agreement table, the framework seldom returns a positive result for the tables with a sample size greater than or equal to 250 under all the considered table sizes and the true agreement levels.
- The level of false decisions of the framework to detect the grey zones when there is a grey zone in the table is at an acceptable level.
- The location of a grey zone in the agreement table does not impact the accuracy of the proposed framework.
- The real-data examples demonstrate that if a grey zone is detected in the agreement table, it is possible to report a higher magnitude of agreement with high confidence. In that sense, if a practitioner is suspected of a grey zone, such as in the first example, the use of the proposed framework leads to more accurate conclusions.
- Overall, the proposed metric $\Delta$ and its threshold $\tau _{\Delta }$ provide the researchers with an easy to implement, reliable, and accurate way of testing the existence of a grey zone in an agreement table.

A future direction for this research is to extend the definition of grey zones to include attributes of the rating mechanisms other than human assessors, as mentioned in the Discussion Section.

## Supplementary Information

**Additional file 1.** Electronic Supplementary Material for 'Detection of Grey Zones in Inter-rater Agreement Studies

## Data Availability

The datasets used and/or analysed during the current study are available from the corresponding author on reasonable request.
