# Supplementary material for: Detection of grey zones in inter-rater agreement studies
Source: BMC Med Res Methodol. 2023 Jan 5;23:3. doi: 10.1186/s12874-022-01759-7 (PMC9814438; doi:10.1186/s12874-022-01759-7)
Supplement: Supplementary file 1 — Additional file 1. Electronic Supplementary Material for 'Detection of Grey Zones in Inter-rater Agreement Studies [file 12874_2022_1759_MOESM1_ESM.pdf]

Electronic Supplementary Material for 'Detection of Grey Zones in Inter-rater Agreement Studies'

Haydar Demirhan and Ezgi Yilmaz
haydar.demirhan@rmit.edu.au;
ezgiyilmaz@hacettepe.edu.tr

Table S1 Descriptive statistics of κ and Δ calculated for (ρ, n) pairs.

		κ			Δ				
ρ	n	Min	Med	Max	Min	Med	90th	95th	Max
0.45	50	-0.125	0.209	0.539	3.861	27.308	291.607	291.607	312.726
0.45	100	-0.002	0.213	0.442	4.564	16.053	246.335	413.388	413.388
0.45	200	0.049	0.210	0.388	5.339	12.830	55.564	55.564	247.037
0.45	300	0.053	0.209	0.364	5.279	12.167	17.114	17.114	268.517
0.45	400	0.085	0.206	0.322	6.526	11.972	33.634	33.634	51.478
0.45	500	0.100	0.207	0.308	6.157	11.508	15.630	15.863	35.401
0.45	1000	0.134	0.209	0.297	6.790	10.907	14.185	14.185	25.180
0.50	50	-0.163	0.230	0.601	2.939	21.067	223.222	223.222	424.269
0.50	100	0.008	0.231	0.444	4.585	13.777	376.726	376.726	376.726
0.50	200	0.026	0.236	0.397	5.169	11.030	24.614	24.614	332.055
0.50	300	0.100	0.235	0.360	5.464	10.433	25.308	25.308	35.637
0.50	400	0.122	0.238	0.353	5.404	9.846	13.630	13.630	31.639
0.50	500	0.113	0.236	0.346	5.715	9.912	12.526	12.526	27.523
0.50	1000	0.168	0.236	0.302	6.050	9.497	12.310	12.310	18.969
0.55	50	-0.113	0.253	0.630	2.761	16.248	197.758	240.713	265.199
0.55	100	0.053	0.263	0.497	3.998	10.750	158.032	158.032	249.900
0.55	200	0.108	0.268	0.439	4.456	9.208	17.471	17.471	51.593
0.55	300	0.151	0.266	0.394	5.157	8.907	14.197	14.197	27.817
0.55	400	0.154	0.268	0.390	4.720	8.766	10.255	10.255	20.651
0.55	500	0.169	0.267	0.367	4.794	8.488	8.778	8.778	20.789
0.55	1000	0.184	0.268	0.346	5.434	8.300	11.714	11.714	15.273
0.60	50	-0.065	0.298	0.569	3.437	11.366	154.520	154.520	280.281
0.60	100	0.068	0.292	0.525	3.792	9.013	43.027	104.885	181.672
0.60	200	0.144	0.299	0.466	4.170	7.994	13.187	13.187	22.728
0.60	300	0.147	0.302	0.471	4.167	7.658	8.497	8.497	20.729
0.60	400	0.162	0.298	0.443	4.475	7.640	12.981	12.981	18.195
0.60	500	0.192	0.300	0.407	4.682	7.573	10.468	10.468	15.085
0.60	1000	0.208	0.300	0.380	4.603	7.492	9.072	9.072	15.480
0.65	50	-0.028	0.332	0.635	2.543	9.014	197.864	197.864	425.251
0.65	100	0.082	0.331	0.591	3.702	7.416	16.878	16.878	146.471
0.65	200	0.120	0.332	0.496	3.988	6.979	7.376	13.104	28.008
0.65	300	0.203	0.335	0.479	4.037	6.827	7.443	7.443	14.356
0.65	400	0.205	0.335	0.476	4.061	6.751	8.806	8.806	13.703
0.65	500	0.225	0.335	0.441	4.008	6.678	7.735	7.735	15.502
0.65	1000	0.249	0.339	0.430	4.675	6.596	8.962	8.962	12.296
0.70	50	0.054	0.365	0.660	2.541	7.249	39.341	39.341	592.563
0.70	100	0.142	0.371	0.621	2.852	6.363	10.605	14.711	46.144
0.70	200	0.234	0.375	0.538	3.580	6.061	7.004	7.004	12.723
0.70	300	0.240	0.375	0.506	3.647	5.990	7.405	7.405	12.772
0.70	400	0.275	0.374	0.503	3.711	5.969	6.721	6.721	11.023
0.70	500	0.269	0.378	0.492	3.918	5.899	8.601	8.601	11.609
0.70	1000	0.298	0.374	0.452	3.752	5.800	7.963	7.963	10.539
0.75	50	0.012	0.419	0.728	1.485	5.976	38.841	38.841	146.074
0.75	100	0.157	0.418	0.635	2.288	5.438	6.938	6.938	29.757
0.75	200	0.232	0.418	0.586	3.173	5.267	6.729	6.729	11.456
0.75	300	0.268	0.420	0.548	3.358	5.153	5.788	5.788	11.947
0.75	400	0.286	0.421	0.547	3.379	5.226	5.501	7.067	9.048
0.75	500	0.313	0.418	0.552	3.347	5.227	7.050	7.050	9.328
0.75	1000	0.336	0.421	0.485	3.679	5.133	5.747	5.747	9.200
0.80	50	0.102	0.459	0.784	1.670	4.950	8.300	21.983	393.512
0.80	100	0.253	0.471	0.771	1.456	4.690	5.238	5.298	12.260
0.80	200	0.316	0.466	0.609	2.823	4.504	4.887	4.887	9.829
0.80	300	0.355	0.470	0.593	3.020	4.512	4.996	4.996	10.480
0.80	400	0.364	0.473	0.589	3.154	4.497	4.594	5.392	8.911
0.80	500	0.372	0.472	0.591	3.068	4.562	5.247	5.643	7.844
0.80	1000	0.399	0.473	0.551	3.170	4.619	4.660	4.660	8.635
0.85	50	0.074	0.531	0.816	1.516	3.872	6.317	6.317	808.094
0.85	100	0.332	0.532	0.771	1.385	3.878	4.630	4.630	8.992
0.85	200	0.387	0.532	0.722	2.327	3.839	4.600	4.600	7.864
0.85	300	0.396	0.534	0.664	2.524	3.926	4.948	4.948	6.930
0.85	400	0.400	0.534	0.646	2.405	3.914	4.234	4.234	7.408
0.85	500	0.432	0.535	0.635	2.814	3.893	4.041	4.041	6.056
0.85	1000	0.467	0.534	0.604	2.787	4.008	3.836	3.913	6.486
0.90	50	0.242	0.605	0.876	1.336	3.153	4.274	4.274	59.505
0.90	100	0.410	0.607	0.831	1.593	3.203	4.349	4.349	7.542
0.90	200	0.470	0.608	0.742	1.955	3.274	4.076	4.076	6.601
0.90	300	0.498	0.614	0.733	2.088	3.280	3.293	3.293	6.022
0.90	400	0.524	0.612	0.705	2.150	3.273	4.288	4.288	5.839
0.90	500	0.504	0.614	0.701	2.105	3.281	5.081	5.081	6.507
0.90	1000	0.541	0.613	0.676	2.410	3.321	3.775	3.775	5.933

Table S2 Sample size, correlation coefficient, true kappa, counts of true and false classifications, accuracy measures, cell probabilities used to inject grey zone and the corresponding kappa after injecting a grey zone in the table for 3x3 agreement tables.

											Cell probabilities to create GZ									Corresponding
**Case**	**n**	**rho**	**True kappa**	**GZ+ TGZ+**	**GZ- TGZ+**	**GZ+ TGZ-**	**GZ- TGZ-**	**Sensitivity**	**Specificity**	**MCC**	**(1,1)**	**(1,2)**	**(1,3)**	**(2,1)**	**(2,2)**	**(2,3)**	**(3,1)**	**(3,2)**	**(3,3)**	**Kappa under GZ**
	50	0.960	0.639	9126	874	385	9615	0.913	0.962	0.875	0.1901	**0.2502**	0.0001	0.0031	0.2501	0.0051	0.0001	0.0001	0.3001	0.626
	50	0.980	0.756	7352	2648	58	9942	0.735	0.994	0.755	0.2451	**0.1862**	0.0001	0.0001	0.2581	0.0001	0.0001	0.0001	0.3101	0.727
	50	0.986	0.817	4017	5983	80	9920	0.402	0.992	0.488	0.1600	**0.1400**	0.0001	0.0001	0.3500	0.0001	0.0001	0.0001	0.3495	0.786
	100	0.930	0.639	9906	94	168	9832	0.991	0.983	0.974	0.2361	**0.1702**	0.0001	0.0031	0.2051	0.0051	0.0001	0.0801	0.3001	0.625
	100	0.965	0.744	8432	1568	98	9902	0.843	0.990	0.843	0.2961	**0.1102**	0.0001	0.0031	0.2051	0.0051	0.0001	0.0501	0.3301	0.749
	100	0.985	0.835	9338	662	749	9251	0.934	0.925	0.859	0.2961	**0.1102**	0.0001	0.0001	0.2131	0.0001	0.0001	0.0010	0.3801	0.834
**Cell (1,2)**	250	0.925	0.633	10000	0	777	9223	1.000	0.922	0.925	0.2091	**0.1971**	0.0001	0.0081	0.2361	0.0201	0.0001	0.0261	0.3041	0.632
**creates**	250	0.963	0.753	9973	27	2141	7859	0.997	0.786	0.801	0.2091	**0.1101**	0.0001	0.0081	0.3231	0.0201	0.0001	0.0261	0.3041	0.753
**GZ**	250	0.977	0.838	10000	0	3288	6712	1.000	0.671	0.711	0.2091	**0.1101**	0.0001	0.0001	0.3511	0.0001	0.0001	0.0001	0.3301	0.834
	500	0.910	0.630	10000	0	846	9154	1.000	0.915	0.919	0.2434	**0.1468**	0.0001	0.0221	0.1941	0.0361	0.0041	0.0401	0.3141	0.631
	500	0.960	0.754	10000	0	733	9267	1.000	0.927	0.929	0.2634	**0.1268**	0.0001	0.0021	0.2341	0.0161	0.0041	0.0201	0.3341	0.750
	500	0.984	0.838	10000	0	2225	7775	1.000	0.778	0.797	0.2794	**0.1108**	0.0001	0.0021	0.2541	0.0001	0.0001	0.0001	0.3541	0.833
	1000	0.900	0.632	10000	0	1424	8576	1.000	0.858	0.866	0.2293	**0.1139**	0.0001	0.0281	0.2081	0.0601	0.0011	0.0401	0.3201	0.636
	1000	0.960	0.767	10000	0	1430	8570	1.000	0.857	0.866	0.2593	**0.0839**	0.0001	0.0201	0.2561	0.0201	0.0011	0.0401	0.3201	0.754
	1000	0.980	0.832	10000	0	212	9788	1.000	0.979	0.979	0.2493	**0.0939**	0.0001	0.0201	0.2761	0.0201	0.0011	0.0001	0.3601	0.828
	50	0.960	0.639	9127	873	385	9615	0.913	0.962	0.875	0.1901	0.0031	0.0001	**0.2502**	0.2501	0.0051	0.0001	0.0001	0.3001	0.626
	50	0.980	0.756	7365	2635	58	9942	0.737	0.994	0.756	0.2451	0.0001	0.0001	**0.1862**	0.2581	0.0001	0.0001	0.0001	0.3101	0.727
	50	0.986	0.817	3999	6001	80	9920	0.400	0.992	0.486	0.1600	0.0001	0.0001	**0.1400**	0.3500	0.0001	0.0001	0.0001	0.3495	0.786
	100	0.930	0.639	9895	105	168	9832	0.990	0.983	0.973	0.2361	0.0031	0.0001	**0.1702**	0.2051	0.0051	0.0001	0.0801	0.3001	0.618
	100	0.965	0.744	8456	1544	98	9902	0.846	0.990	0.845	0.2961	0.0031	0.0001	**0.1102**	0.2051	0.0051	0.0001	0.0501	0.3301	0.749
	100	0.985	0.835	9308	692	749	9251	0.931	0.925	0.856	0.2961	0.0001	0.0001	**0.1102**	0.2131	0.0001	0.0001	0.0010	0.3801	0.834
**Cell (2,1)**	250	0.925	0.633	10000	0	777	9223	1.000	0.922	0.925	0.2091	0.0081	0.0001	**0.1971**	0.2361	0.0201	0.0001	0.0261	0.3041	0.632
**creates**	250	0.963	0.753	9973	27	2118	7882	0.997	0.788	0.803	0.2091	0.0081	0.0001	**0.1101**	0.3231	0.0201	0.0001	0.0261	0.3041	0.753
**GZ**	250	0.977	0.838	10000	0	3362	6638	1.000	0.664	0.705	0.2091	0.0001	0.0001	**0.1101**	0.3511	0.0001	0.0001	0.0001	0.3301	0.834
	500	0.910	0.630	10000	0	811	9189	1.000	0.919	0.922	0.2434	0.0221	0.0001	**0.1468**	0.1941	0.0361	0.0041	0.0401	0.3141	0.631
	500	0.960	0.754	10000	0	729	9271	1.000	0.927	0.930	0.2634	0.0021	0.0001	**0.1268**	0.2341	0.0161	0.0041	0.0201	0.3341	0.750
	500	0.984	0.838	10000	0	2287	7713	1.000	0.771	0.792	0.2794	0.0021	0.0001	**0.1108**	0.2541	0.0001	0.0001	0.0001	0.3541	0.833
	1000	0.900	0.632	10000	0	222	9778	1.000	0.978	0.978	0.2293	0.0281	0.0001	**0.1139**	0.2081	0.0601	0.0011	0.0401	0.3201	0.637
	1000	0.960	0.767	10000	0	1471	8529	1.000	0.853	0.862	0.2593	0.0201	0.0001	**0.0839**	0.2561	0.0201	0.0011	0.0401	0.3201	0.753
	1000	0.980	0.832	10000	0	215	9785	1.000	0.979	0.979	0.2493	0.0201	0.0001	**0.0939**	0.2761	0.0201	0.0011	0.0001	0.3601	0.829
	50	0.960	0.639	9379	621	385	9615	0.938	0.962	0.900	0.1901	0.0031	0.0001	0.0051	0.2501	**0.2402**	0.0001	0.0001	0.3101	0.625
	50	0.980	0.756	7400	2600	58	9942	0.740	0.994	0.759	0.2451	0.0001	0.0001	0.0001	0.2581	**0.1862**	0.0001	0.0001	0.3101	0.722
	50	0.986	0.817	4024	5976	80	9920	0.402	0.992	0.488	0.1600	0.0001	0.0001	0.0001	0.3500	**0.1400**	0.0001	0.0001	0.3495	0.778
	100	0.930	0.639	9903	97	168	9832	0.990	0.983	0.974	0.2361	0.0701	0.0001	0.0051	0.2051	**0.1702**	0.0001	0.0031	0.3101	0.629
	100	0.965	0.744	8357	1643	98	9902	0.836	0.990	0.836	0.2961	0.0501	0.0001	0.0051	0.2051	**0.1102**	0.0001	0.0031	0.3301	0.747
	100	0.985	0.835	9097	903	749	9251	0.910	0.925	0.835	0.2961	0.0001	0.0001	0.0001	0.2131	**0.1102**	0.0001	0.0010	0.3801	0.831
**Cell (2,3)**	250	0.925	0.633	10000	0	771	9229	1.000	0.923	0.926	0.2091	0.0261	0.0001	0.0201	0.2361	**0.1971**	0.0001	0.0081	0.3041	0.624
**creates**	250	0.963	0.753	9970	30	2146	7854	0.997	0.785	0.801	0.2091	0.0081	0.0001	0.0201	0.3231	**0.1101**	0.0001	0.0261	0.3041	0.750
**GZ**	250	0.977	0.838	10000	0	3389	6611	1.000	0.661	0.703	0.2091	0.0001	0.0001	0.0001	0.3511	**0.1101**	0.0001	0.0001	0.3301	0.831
	500	0.910	0.630	10000	0	831	9169	1.000	0.917	0.920	0.2434	0.0401	0.0001	0.0361	0.1941	**0.1468**	0.0041	0.0221	0.3141	0.627
	500	0.960	0.754	10000	0	774	9226	1.000	0.923	0.925	0.2634	0.0021	0.0001	0.0161	0.2341	**0.1268**	0.0041	0.0201	0.3341	0.747
	500	0.984	0.838	10000	0	2331	7669	1.000	0.767	0.789	0.2794	0.0021	0.0001	0.0001	0.2541	**0.1108**	0.0001	0.0001	0.3541	0.831
	1000	0.900	0.632	10000	0	269	9731	1.000	0.973	0.973	0.2293	0.0401	0.0001	0.0601	0.2081	**0.1139**	0.0011	0.0281	0.3201	0.635
	1000	0.960	0.767	10000	0	1509	8491	1.000	0.849	0.859	0.2593	0.0201	0.0001	0.0201	0.2561	**0.0839**	0.0011	0.0401	0.3201	0.753
	1000	0.980	0.832	10000	0	229	9771	1.000	0.977	0.977	0.2493	0.0201	0.0001	0.0201	0.2761	**0.0939**	0.0011	0.0001	0.3601	0.827
	50	0.960	0.639	9349	651	385	9615	0.935	0.962	0.897	0.1901	0.0031	0.0001	0.0051	0.2501	0.0001	0.0001	**0.2402**	0.3101	0.625
	50	0.980	0.756	7292	2708	58	9942	0.729	0.994	0.750	0.2451	0.0001	0.0001	0.0001	0.2581	0.0001	0.0001	**0.1862**	0.3101	0.722
	50	0.986	0.817	3897	6103	80	9920	0.390	0.992	0.478	0.1600	0.0001	0.0001	0.0001	0.3500	0.0001	0.0001	**0.1400**	0.3495	0.778
	100	0.930	0.639	9893	107	168	9832	0.989	0.983	0.973	0.2361	0.0701	0.0001	0.0051	0.2051	0.0031	0.0001	**0.1702**	0.3101	0.635
	100	0.965	0.744	8373	1627	98	9902	0.837	0.990	0.837	0.2961	0.0501	0.0001	0.0051	0.2051	0.0031	0.0001	**0.1102**	0.3301	0.749
	100	0.985	0.835	9021	979	749	9251	0.902	0.925	0.827	0.2961	0.0001	0.0001	0.0001	0.2131	0.0010	0.0001	**0.1102**	0.3801	0.831
**Cell (3,2)**	250	0.925	0.633	10000	0	792	9208	1.000	0.921	0.924	0.2091	0.0261	0.0001	0.0201	0.2361	0.0081	0.0001	**0.1971**	0.3041	0.625
**creates**	250	0.963	0.753	9970	30	2097	7903	0.997	0.790	0.805	0.2091	0.0081	0.0001	0.0201	0.3231	0.0261	0.0001	**0.1101**	0.3041	0.750
**GZ**	250	0.977	0.838	10000	0	3334	6666	1.000	0.667	0.707	0.2091	0.0001	0.0001	0.0001	0.3511	0.0001	0.0001	**0.1101**	0.3301	0.831
	500	0.910	0.630	10000	0	782	9218	1.000	0.922	0.925	0.2434	0.0401	0.0001	0.0361	0.1941	0.0221	0.0041	**0.1468**	0.3141	0.628
	500	0.960	0.754	10000	0	786	9214	1.000	0.921	0.924	0.2634	0.0021	0.0001	0.0161	0.2341	0.0201	0.0041	**0.1268**	0.3341	0.748
	500	0.984	0.838	10000	0	2314	7686	1.000	0.769	0.790	0.2794	0.0021	0.0001	0.0001	0.2541	0.0001	0.0001	**0.1108**	0.3541	0.831
	1000	0.900	0.632	10000	0	271	9729	1.000	0.973	0.973	0.2293	0.0401	0.0001	0.0601	0.2081	0.0281	0.0011	**0.1139**	0.3201	0.634
	1000	0.960	0.767	10000	0	1404	8596	1.000	0.860	0.868	0.2593	0.0201	0.0001	0.0201	0.2561	0.0401	0.0011	**0.0839**	0.3201	0.753
	1000	0.980	0.832	10000	0	229	9771	1.000	0.977	0.977	0.2493	0.0201	0.0001	0.0001	0.2761	0.0201	0.0011	**0.0939**	0.3601	0.826

Table S3 Sample size, correlation coefficient, true kappa, counts of true and false classifications, accuracy measures, cell probabilities used to inject grey zone and the corresponding kappa after injecting a grey zone in the table for 4x4 agreement tables.

											Cell probabilities to create GZ																Corresponding
**Case**	**n**	**rho**	**True kappa**	**GZ+ TGZ+**	**GZ- TGZ+**	**GZ+ TGZ-**	**GZ- TGZ-**	**Sensitivity**	**Specificity**	**MCC**	**(1,1)**	**(1,2)**	**(1,3)**	**(1,4)**	**(2,1)**	**(2,2)**	**(2,3)**	**(2,4)**	**(3,1)**	**(3,2)**	**(3,3)**	**(3,4)**	**(4,1)**	**(4,2)**	**(4,3)**	**(4,4)**	**Kappa under GZ**
	**50**	0.911	0.624	3958	6042	72	9928	0.396	0.993	0.484	0.1354	**0.1678**	0.0005	0.0001	0.0051	0.1431	0.0211	0.0021	0.0021	0.0531	0.1671	0.0181	0.0001	0.0001	0.0151	0.2691	0.626
	50	0.969	0.731	5224	4776	12	9988	0.522	0.999	0.593	0.1354	**0.1678**	0.0005	0.0001	0.0051	0.1731	0.0011	0.0021	0.0021	0.0031	0.2271	0.0081	0.0001	0.0001	0.0051	0.2691	0.743
	50	0.982	0.839	4839	5161	13	9987	0.484	0.999	0.563	0.1524	**0.1500**	0.0001	0.0001	0.0001	0.1809	0.0001	0.0001	0.0001	0.0001	0.2386	0.0001	0.0001	0.0001	0.0001	0.2771	0.801
	100	0.935	0.612	9694	306	993	9007	0.969	0.901	0.872	0.1354	**0.1678**	0.0005	0.0001	0.0051	0.1431	0.0211	0.0021	0.0021	0.0531	0.1671	0.0181	0.0001	0.0001	0.0151	0.2691	0.626
	100	0.982	0.746	9804	196	727	9273	0.980	0.927	0.909	0.1354	**0.1678**	0.0005	0.0001	0.0051	0.1731	0.0011	0.0021	0.0021	0.0031	0.2271	0.0081	0.0001	0.0001	0.0051	0.2691	0.743
	100	0.992	0.840	9959	41	229	9771	0.996	0.977	0.973	0.1524	**0.1500**	0.0001	0.0001	0.0001	0.1809	0.0001	0.0001	0.0001	0.0001	0.2386	0.0001	0.0001	0.0001	0.0001	0.2771	0.801
**Cell (1,2) creates GZ**	250	0.945	0.616	10000	0	933	9067	1.000	0.907	0.911	0.1354	**0.1678**	0.0005	0.0001	0.0051	0.1431	0.0211	0.0021	0.0021	0.0531	0.1671	0.0181	0.0001	0.0001	0.0151	0.2691	0.626
	250	0.975	0.755	10000	0	623	9377	1.000	0.938	0.940	0.1354	**0.1678**	0.0005	0.0001	0.0051	0.1731	0.0011	0.0021	0.0021	0.0031	0.2271	0.0081	0.0001	0.0001	0.0051	0.2691	0.743
	250	0.987	0.824	10000	0	1065	8935	1.000	0.894	0.899	0.1524	**0.1500**	0.0001	0.0001	0.0001	0.1809	0.0001	0.0001	0.0001	0.0001	0.2386	0.0001	0.0001	0.0001	0.0001	0.2771	0.801
	500	0.945	0.613	10000	0	1381	8619	1.000	0.862	0.870	0.1354	**0.1678**	0.0005	0.0001	0.0051	0.1431	0.0211	0.0021	0.0021	0.0531	0.1671	0.0181	0.0001	0.0001	0.0151	0.2691	0.626
	500	0.977	0.747	10000	0	562	9438	1.000	0.944	0.945	0.1354	**0.1678**	0.0005	0.0001	0.0051	0.1731	0.0011	0.0021	0.0021	0.0031	0.2271	0.0081	0.0001	0.0001	0.0051	0.2691	0.743
	500	0.987	0.824	10000	0	522	9478	1.000	0.948	0.949	0.1524	**0.1500**	0.0001	0.0001	0.0001	0.1809	0.0001	0.0001	0.0001	0.0001	0.2386	0.0001	0.0001	0.0001	0.0001	0.2771	0.801
	1000	0.940	0.617	10000	0	757	9243	1.000	0.924	0.927	0.1354	**0.1678**	0.0005	0.0001	0.0051	0.1431	0.0211	0.0021	0.0021	0.0531	0.1671	0.0181	0.0001	0.0001	0.0151	0.2691	0.626
	1000	0.975	0.740	10000	0	1450	8550	1.000	0.855	0.864	0.1354	**0.1678**	0.0005	0.0001	0.0051	0.1731	0.0011	0.0021	0.0021	0.0031	0.2271	0.0081	0.0001	0.0001	0.0051	0.2691	0.743
	1000	0.985	0.828	10000	0	563	9437	1.000	0.944	0.945	0.1524	**0.1500**	0.0001	0.0001	0.0001	0.1809	0.0001	0.0001	0.0001	0.0001	0.2386	0.0001	0.0001	0.0001	0.0001	0.2771	0.801
	50	0.911	0.624	3929	6071	72	9928	0.393	0.993	0.482	0.1354	0.0051	0.0005	0.0001	**0.1678**	0.1431	0.0211	0.0021	0.0021	0.0531	0.1671	0.0181	0.0001	0.0001	0.0151	0.2691	0.626
	50	0.969	0.731	5240	4760	12	9988	0.524	0.999	0.594	0.1354	0.0051	0.0005	0.0001	**0.1678**	0.1731	0.0011	0.0021	0.0021	0.0031	0.2271	0.0081	0.0001	0.0001	0.0051	0.2691	0.743
	50	0.982	0.839	4839	5161	13	9987	0.484	0.999	0.563	0.1524	0.0001	0.0001	0.0001	**0.1500**	0.1809	0.0001	0.0001	0.0001	0.0001	0.2386	0.0001	0.0001	0.0001	0.0001	0.2771	0.801
	100	0.935	0.612	9676	324	993	9007	0.968	0.901	0.870	0.1354	0.0051	0.0005	0.0001	**0.1678**	0.1431	0.0211	0.0021	0.0021	0.0531	0.1671	0.0181	0.0001	0.0001	0.0151	0.2691	0.626
	100	0.982	0.746	9809	191	758	9242	0.981	0.924	0.907	0.1354	0.0051	0.0005	0.0001	**0.1678**	0.1731	0.0011	0.0021	0.0021	0.0031	0.2271	0.0081	0.0001	0.0001	0.0051	0.2691	0.743
	100	0.992	0.840	9949	51	204	9796	0.995	0.980	0.975	0.1524	0.0001	0.0001	0.0001	**0.1500**	0.1809	0.0001	0.0001	0.0001	0.0001	0.2386	0.0001	0.0001	0.0001	0.0001	0.2771	0.801
**Cell (2,1) creates GZ**	250	0.945	0.616	10000	0	910	9090	1.000	0.909	0.913	0.1354	0.0051	0.0005	0.0001	**0.1678**	0.1431	0.0211	0.0021	0.0021	0.0531	0.1671	0.0181	0.0001	0.0001	0.0151	0.2691	0.626
	250	0.975	0.755	10000	0	612	9388	1.000	0.939	0.941	0.1354	0.0051	0.0005	0.0001	**0.1678**	0.1731	0.0011	0.0021	0.0021	0.0031	0.2271	0.0081	0.0001	0.0001	0.0051	0.2691	0.743
	250	0.987	0.824	10000	0	1133	8867	1.000	0.887	0.892	0.1524	0.0001	0.0001	0.0001	**0.1500**	0.1809	0.0001	0.0001	0.0001	0.0001	0.2386	0.0001	0.0001	0.0001	0.0001	0.2771	0.801
	500	0.945	0.613	10000	0	1393	8607	1.000	0.861	0.869	0.1354	0.0051	0.0005	0.0001	**0.1678**	0.1431	0.0211	0.0021	0.0021	0.0531	0.1671	0.0181	0.0001	0.0001	0.0151	0.2691	0.626
	500	0.977	0.747	10000	0	572	9428	1.000	0.943	0.944	0.1354	0.0051	0.0005	0.0001	**0.1678**	0.1731	0.0011	0.0021	0.0021	0.0031	0.2271	0.0081	0.0001	0.0001	0.0051	0.2691	0.743
	500	0.987	0.824	10000	0	484	9516	1.000	0.952	0.953	0.1524	0.0001	0.0001	0.0001	**0.1500**	0.1809	0.0001	0.0001	0.0001	0.0001	0.2386	0.0001	0.0001	0.0001	0.0001	0.2771	0.801
	1000	0.940	0.617	10000	0	765	9235	1.000	0.924	0.926	0.1354	0.0051	0.0005	0.0001	**0.1678**	0.1431	0.0211	0.0021	0.0021	0.0531	0.1671	0.0181	0.0001	0.0001	0.0151	0.2691	0.626
	1000	0.975	0.740	10000	0	1319	8681	1.000	0.868	0.876	0.1354	0.0051	0.0005	0.0001	**0.1678**	0.1731	0.0011	0.0021	0.0021	0.0031	0.2271	0.0081	0.0001	0.0001	0.0051	0.2691	0.743
	1000	0.985	0.828	10000	0	544	9456	1.000	0.946	0.947	0.1524	0.0001	0.0001	0.0001	**0.1500**	0.1809	0.0001	0.0001	0.0001	0.0001	0.2386	0.0001	0.0001	0.0001	0.0001	0.2771	0.801
	50	0.911	0.624	746	9254	72	9928	0.075	0.993	0.170	0.1354	0.0051	0.0005	0.0001	0.0211	0.1431	**0.1678**	0.0021	0.0021	0.0531	0.1671	0.0181	0.0001	0.0001	0.0151	0.2691	0.626
	50	0.969	0.731	5534	4466	12	9988	0.553	0.999	0.617	0.1354	0.0051	0.0005	0.0001	0.0011	0.1731	**0.1678**	0.0021	0.0021	0.0031	0.2271	0.0081	0.0001	0.0001	0.0051	0.2691	0.743
	50	0.982	0.839	4836	5164	13	9987	0.484	0.999	0.563	0.1524	0.0001	0.0001	0.0001	0.0001	0.1809	**0.1500**	0.0001	0.0001	0.0001	0.2386	0.0001	0.0001	0.0001	0.0001	0.2771	0.801
	100	0.935	0.612	3236	6764	993	9007	0.324	0.901	0.275	0.1354	0.0051	0.0005	0.0001	0.0211	0.1431	**0.1678**	0.0021	0.0021	0.0531	0.1671	0.0181	0.0001	0.0001	0.0151	0.2691	0.626
	100	0.982	0.746	9909	91	746	9254	0.991	0.925	0.918	0.1354	0.0051	0.0005	0.0001	0.0011	0.1731	**0.1678**	0.0021	0.0021	0.0031	0.2271	0.0081	0.0001	0.0001	0.0051	0.2691	0.743
	100	0.992	0.840	9946	54	220	9780	0.995	0.978	0.973	0.1524	0.0001	0.0001	0.0001	0.0001	0.1809	**0.1500**	0.0001	0.0001	0.0001	0.2386	0.0001	0.0001	0.0001	0.0001	0.2771	0.801
**Cell (2,3) creates GZ**	250	0.945	0.616	8792	1208	903	9097	0.879	0.910	0.789	0.1354	0.0051	0.0005	0.0001	0.0211	0.1431	**0.1678**	0.0021	0.0021	0.0531	0.1671	0.0181	0.0001	0.0001	0.0151	0.2691	0.626
	250	0.975	0.755	10000	0	611	9389	1.000	0.939	0.941	0.1354	0.0051	0.0005	0.0001	0.0011	0.1731	**0.1678**	0.0021	0.0021	0.0031	0.2271	0.0081	0.0001	0.0001	0.0051	0.2691	0.743
	250	0.987	0.824	10000	0	1102	8898	1.000	0.890	0.895	0.1524	0.0001	0.0001	0.0001	0.0001	0.1809	**0.1500**	0.0001	0.0001	0.0001	0.2386	0.0001	0.0001	0.0001	0.0001	0.2771	0.801
	500	0.945	0.613	9993	7	1402	8598	0.999	0.860	0.868	0.1354	0.0051	0.0005	0.0001	0.0211	0.1431	**0.1678**	0.0021	0.0021	0.0531	0.1671	0.0181	0.0001	0.0001	0.0151	0.2691	0.626
	500	0.977	0.747	10000	0	559	9441	1.000	0.944	0.946	0.1354	0.0051	0.0005	0.0001	0.0011	0.1731	**0.1678**	0.0021	0.0021	0.0031	0.2271	0.0081	0.0001	0.0001	0.0051	0.2691	0.743
	500	0.987	0.824	10000	0	527	9473	1.000	0.947	0.949	0.1524	0.0001	0.0001	0.0001	0.0001	0.1809	**0.1500**	0.0001	0.0001	0.0001	0.2386	0.0001	0.0001	0.0001	0.0001	0.2771	0.801
	1000	0.940	0.617	10000	0	740	9260	1.000	0.926	0.929	0.1354	0.0051	0.0005	0.0001	0.0211	0.1431	**0.1678**	0.0021	0.0021	0.0531	0.1671	0.0181	0.0001	0.0001	0.0151	0.2691	0.626
	1000	0.975	0.740	10000	0	1381	8619	1.000	0.862	0.870	0.1354	0.0051	0.0005	0.0001	0.0011	0.1731	**0.1678**	0.0021	0.0021	0.0031	0.2271	0.0081	0.0001	0.0001	0.0051	0.2691	0.743
	1000	0.985	0.828	10000	0	572	9428	1.000	0.943	0.944	0.1524	0.0001	0.0001	0.0001	0.0001	0.1809	**0.1500**	0.0001	0.0001	0.0001	0.2386	0.0001	0.0001	0.0001	0.0001	0.2771	0.801
	50	0.911	0.624	719	9281	72	9928	0.072	0.993	0.166	0.1354	0.0051	0.0005	0.0001	0.0211	0.1431	0.0531	0.0021	0.0021	**0.1678**	0.1671	0.0181	0.0001	0.0001	0.0151	0.2691	0.626
	50	0.969	0.731	5527	4473	12	9988	0.553	0.999	0.616	0.1354	0.0051	0.0005	0.0001	0.0011	0.1731	0.0031	0.0021	0.0021	**0.1678**	0.2271	0.0081	0.0001	0.0001	0.0051	0.2691	0.743
	50	0.982	0.839	4807	5193	13	9987	0.481	0.999	0.560	0.1524	0.0001	0.0001	0.0001	0.0001	0.1809	0.0001	0.0001	0.0001	**0.1500**	0.2386	0.0001	0.0001	0.0001	0.0001	0.2771	0.801
	100	0.935	0.612	3133	6867	993	9007	0.313	0.901	0.264	0.1354	0.0051	0.0005	0.0001	0.0211	0.1431	0.0531	0.0021	0.0021	**0.1678**	0.1671	0.0181	0.0001	0.0001	0.0151	0.2691	0.626
	100	0.982	0.746	9897	103	752	9248	0.990	0.925	0.916	0.1354	0.0051	0.0005	0.0001	0.0011	0.1731	0.0031	0.0021	0.0021	**0.1678**	0.2271	0.0081	0.0001	0.0001	0.0051	0.2691	0.743
	100	0.992	0.840	9945	55	249	9751	0.995	0.975	0.970	0.1524	0.0001	0.0001	0.0001	0.0001	0.1809	0.0001	0.0001	0.0001	**0.1500**	0.2386	0.0001	0.0001	0.0001	0.0001	0.2771	0.801
**Cell (3,2) creates GZ**	250	0.945	0.616	8734	1266	915	9085	0.873	0.909	0.782	0.1354	0.0051	0.0005	0.0001	0.0211	0.1431	0.0531	0.0021	0.0021	**0.1678**	0.1671	0.0181	0.0001	0.0001	0.0151	0.2691	0.626
	250	0.975	0.755	10000	0	619	9381	1.000	0.938	0.940	0.1354	0.0051	0.0005	0.0001	0.0011	0.1731	0.0031	0.0021	0.0021	**0.1678**	0.2271	0.0081	0.0001	0.0001	0.0051	0.2691	0.743
	250	0.987	0.824	10000	0	1064	8936	1.000	0.894	0.899	0.1524	0.0001	0.0001	0.0001	0.0001	0.1809	0.0001	0.0001	0.0001	**0.1500**	0.2386	0.0001	0.0001	0.0001	0.0001	0.2771	0.801
	500	0.945	0.613	9989	11	1311	8689	0.999	0.869	0.875	0.1354	0.0051	0.0005	0.0001	0.0211	0.1431	0.0531	0.0021	0.0021	**0.1678**	0.1671	0.0181	0.0001	0.0001	0.0151	0.2691	0.626
	500	0.977	0.747	10000	0	541	9459	1.000	0.946	0.947	0.1354	0.0051	0.0005	0.0001	0.0011	0.1731	0.0031	0.0021	0.0021	**0.1678**	0.2271	0.0081	0.0001	0.0001	0.0051	0.2691	0.743
	500	0.987	0.824	10000	0	477	9523	1.000	0.952	0.953	0.1524	0.0001	0.0001	0.0001	0.0001	0.1809	0.0001	0.0001	0.0001	**0.1500**	0.2386	0.0001	0.0001	0.0001	0.0001	0.2771	0.801
	1000	0.940	0.617	10000	0	711	9289	1.000	0.929	0.931	0.1354	0.0051	0.0005	0.0001	0.0211	0.1431	0.0531	0.0021	0.0021	**0.1678**	0.1671	0.0181	0.0001	0.0001	0.0151	0.2691	0.626
	1000	0.975	0.740	10000	0	1372	8628	1.000	0.863	0.871	0.1354	0.0051	0.0005	0.0001	0.0011	0.1731	0.0031	0.0021	0.0021	**0.1678**	0.2271	0.0081	0.0001	0.0001	0.0051	0.2691	0.743
	1000	0.985	0.828	10000	0	518	9482	1.000	0.948	0.949	0.1524	0.0001	0.0001	0.0001	0.0001	0.1809	0.0001	0.0001	0.0001	**0.1500**	0.2386	0.0001	0.0001	0.0001	0.0001	0.2771	0.801
	50	0.911	0.624	2590	7410	72	9928	0.259	0.993	0.371	0.1354	0.0051	0.0005	0.0001	0.0211	0.1431	0.0531	0.0021	0.0021	0.0181	0.1671	**0.1678**	0.0001	0.0001	0.0151	0.2691	0.626
	50	0.969	0.731	5131	4869	12	9988	0.513	0.999	0.586	0.1354	0.0051	0.0005	0.0001	0.0011	0.1731	0.0031	0.0021	0.0021	0.0081	0.2271	**0.1678**	0.0001	0.0001	0.0051	0.2691	0.743
	50	0.982	0.839	4772	5228	15	9987	0.477	0.999	0.557	0.1524	0.0001	0.0001	0.0001	0.0001	0.1809	0.0001	0.0001	0.0001	0.0001	0.2386	**0.1500**	0.0001	0.0001	0.0001	0.2771	0.801
	100	0.935	0.612	8463	1537	993	9007	0.846	0.901	0.748	0.1354	0.0051	0.0005	0.0001	0.0211	0.1431	0.0531	0.0021	0.0021	0.0181	0.1671	**0.1678**	0.0001	0.0001	0.0151	0.2691	0.626
	100	0.982	0.746	9768	232	798	9202	0.977	0.920	0.898	0.1354	0.0051	0.0005	0.0001	0.0011	0.1731	0.0031	0.0021	0.0021	0.0081	0.2271	**0.1678**	0.0001	0.0001	0.0051	0.2691	0.743
	100	0.992	0.840	9948	52	227	9773	0.995	0.977	0.972	0.1524	0.0001	0.0001	0.0001	0.0001	0.1809	0.0001	0.0001	0.0001	0.0001	0.2386	**0.1500**	0.0001	0.0001	0.0001	0.2771	0.801
**Cell (3,4) creates GZ**	250	0.945	0.616	9997	3	901	9099	1.000	0.910	0.913	0.1354	0.0051	0.0005	0.0001	0.0211	0.1431	0.0531	0.0021	0.0021	0.0181	0.1671	**0.1678**	0.0001	0.0001	0.0151	0.2691	0.626
	250	0.975	0.755	10000	0	611	9389	1.000	0.939	0.941	0.1354	0.0051	0.0005	0.0001	0.0011	0.1731	0.0031	0.0021	0.0021	0.0081	0.2271	**0.1678**	0.0001	0.0001	0.0051	0.2691	0.743
	250	0.987	0.824	10000	0	1075	8925	1.000	0.893	0.898	0.1524	0.0001	0.0001	0.0001	0.0001	0.1809	0.0001	0.0001	0.0001	0.0001	0.2386	**0.1500**	0.0001	0.0001	0.0001	0.2771	0.801
	500	0.945	0.613	10000	0	1367	8633	1.000	0.863	0.871	0.1354	0.0051	0.0005	0.0001	0.0211	0.1431	0.0531	0.0021	0.0021	0.0181	0.1671	**0.1678**	0.0001	0.0001	0.0151	0.2691	0.626
	500	0.977	0.747	10000	0	572	9428	1.000	0.943	0.944	0.1354	0.0051	0.0005	0.0001	0.0011	0.1731	0.0031	0.0021	0.0021	0.0081	0.2271	**0.1678**	0.0001	0.0001	0.0051	0.2691	0.743
	500	0.987	0.824	10000	0	530	9470	1.000	0.947	0.948	0.1524	0.0001	0.0001	0.0001	0.0001	0.1809	0.0001	0.0001	0.0001	0.0001	0.2386	**0.1500**	0.0001	0.0001	0.0001	0.2771	0.801
	1000	0.940	0.617	10000	0	720	9280	1.000	0.928	0.930	0.1354	0.0051	0.0005	0.0001	0.0211	0.1431	0.0531	0.0021	0.0021	0.0181	0.1671	**0.1678**	0.0001	0.0001	0.0151	0.2691	0.626
	1000	0.975	0.740	10000	0	1386	8614	1.000	0.861	0.870	0.1354	0.0051	0.0005	0.0001	0.0011	0.1731	0.0031	0.0021	0.0021	0.0081	0.2271	**0.1678**	0.0001	0.0001	0.0051	0.2691	0.743
	1000	0.985	0.828	10000	0	545	9455	1.000	0.946	0.947	0.1524	0.0001	0.0001	0.0001	0.0001	0.1809	0.0001	0.0001	0.0001	0.0001	0.2386	**0.1500**	0.0001	0.0001	0.0001	0.2771	0.801
	50	0.911	0.624	2617	7383	72	9928	0.262	0.993	0.373	0.1354	0.0051	0.0005	0.0001	0.0211	0.1431	0.0531	0.0021	0.0021	0.0181	0.1671	0.0151	0.0001	0.0001	**0.1678**	0.2691	0.626
	50	0.969	0.731	5082	4918	12	9988	0.508	0.999	0.582	0.1354	0.0051	0.0005	0.0001	0.0011	0.1731	0.0031	0.0021	0.0021	0.0081	0.2271	0.0051	0.0001	0.0001	**0.1678**	0.2691	0.743
	50	0.982	0.839	4742	5258	13	9987	0.474	0.999	0.555	0.1524	0.0001	0.0001	0.0001	0.0001	0.1809	0.0001	0.0001	0.0001	0.0001	0.2386	0.0001	0.0001	0.0001	**0.1500**	0.2771	0.801
	100	0.935	0.612	8555	1445	993	9007	0.856	0.901	0.757	0.1354	0.0051	0.0005	0.0001	0.0211	0.1431	0.0531	0.0021	0.0021	0.0181	0.1671	0.0151	0.0001	0.0001	**0.1678**	0.2691	0.626
	100	0.982	0.746	9791	209	798	9202	0.979	0.920	0.901	0.1354	0.0051	0.0005	0.0001	0.0011	0.1731	0.0031	0.0021	0.0021	0.0081	0.2271	0.0051	0.0001	0.0001	**0.1678**	0.2691	0.743
	100	0.992	0.840	9946	54	227	9773	0.995	0.977	0.972	0.1524	0.0001	0.0001	0.0001	0.0001	0.1809	0.0001	0.0001	0.0001	0.0001	0.2386	0.0001	0.0001	0.0001	**0.1500**	0.2771	0.801
**Cell (4,3) creates GZ**	250	0.945	0.616	9998	2	892	9108	1.000	0.911	0.914	0.1354	0.0051	0.0005	0.0001	0.0211	0.1431	0.0531	0.0021	0.0021	0.0181	0.1671	0.0151	0.0001	0.0001	**0.1678**	0.2691	0.626
	250	0.975	0.755	10000	0	643	9357	1.000	0.936	0.938	0.1354	0.0051	0.0005	0.0001	0.0011	0.1731	0.0031	0.0021	0.0021	0.0081	0.2271	0.0051	0.0001	0.0001	**0.1678**	0.2691	0.743
	250	0.987	0.824	10000	0	1111	8889	1.000	0.889	0.894	0.1524	0.0001	0.0001	0.0001	0.0001	0.1809	0.0001	0.0001	0.0001	0.0001	0.2386	0.0001	0.0001	0.0001	**0.1500**	0.2771	0.801
	500	0.945	0.613	10000	0	1368	8632	1.000	0.863	0.871	0.1354	0.0051	0.0005	0.0001	0.0211	0.1431	0.0531	0.0021	0.0021	0.0181	0.1671	0.0151	0.0001	0.0001	**0.1678**	0.2691	0.626
	500	0.977	0.747	10000	0	561	9439	1.000	0.944	0.945	0.1354	0.0051	0.0005	0.0001	0.0011	0.1731	0.0031	0.0021	0.0021	0.0081	0.2271	0.0051	0.0001	0.0001	**0.1678**	0.2691	0.743
	500	0.987	0.824	10000	0	505	9495	1.000	0.950	0.951	0.1524	0.0001	0.0001	0.0001	0.0001	0.1809	0.0001	0.0001	0.0001	0.0001	0.2386	0.0001	0.0001	0.0001	**0.1500**	0.2771	0.801
	1000	0.940	0.617	10000	0	712	9288	1.000	0.929	0.931	0.1354	0.0051	0.0005	0.0001	0.0211	0.1431	0.0531	0.0021	0.0021	0.0181	0.1671	0.0151	0.0001	0.0001	**0.1678**	0.2691	0.626
	1000	0.975	0.740	10000	0	1359	8641	1.000	0.864	0.872	0.1354	0.0051	0.0005	0.0001	0.0011	0.1731	0.0031	0.0021	0.0021	0.0081	0.2271	0.0051	0.0001	0.0001	**0.1678**	0.2691	0.743
	1000	0.985	0.828	10000	0	571	9429	1.000	0.943	0.944	0.1524	0.0001	0.0001	0.0001	0.0001	0.1809	0.0001	0.0001	0.0001	0.0001	0.2386	0.0001	0.0001	0.0001	**0.1500**	0.2771	0.801

Suppelementary Codes

Implementation

The following function computes the criterion Δ and the threshold τ_Δ for the detection of grey zones in an input agreement table.

```
detectGreyZone <- function(table){
  require(irrCAC)
  R <- nrow(table)
  if (ncol(table) != R){
    stop("This function requires a square contingency table.")
  }
  n <- sum(table)

  #-------------- Inter-Rater Agreement ------------
  kappa <- kappa2.table(table)
  kappa.linear <- kappa2.table(table, weights = linear.weights(1:R))
  kappa.quadratic <- kappa2.table(table, weights = quadratic.weights(1:R))
  AC2.linear <- gwet.ac1.table(table, weights = linear.weights(1:R))
  AC2.quadratic <- gwet.ac1.table(table, weights = quadratic.weights(1:R))
  BP.linear <- bp2.table(table, weights = linear.weights(1:R))
  BP.quadratic <- bp2.table(table, weights = quadratic.weights(1:R))

  #-------------- Criterion ------------
  expf <- array(0,dim=c(R,R))
  st.res <- array(0,dim=c(R,R))

  for (j in 1:R){
    for (k in 1:R){
      expf[j,k]= (table[j,k]+table[k,j])/2
      st.res[j,k]= (table[j,k]-expf[j,k])/sqrt(expf[j,k])
    }
  }
  st.res[st.res=="NaN"] <- 0

  delta <- st.res/kappa$coeff.val
  delta2 <- max(delta)

  #-------------- Threshold ------------
  tau_Delta <- (-0.0080+0.4090*kappa$coeff.val^{2}+3.331*10^{-5}*n
               -2.467*10^{-8}*n^{2})^{-0.6266}

  #-------------- Result ------------
  if (delta2 > tau_Delta){# Grey zone detected, report AC2 and BP with quadratic weights.
    agreement <- data.frame(Weights = c("Quadratic"),
                            AC2 = AC2.quadratic$coeff.val,
                            BP = BP.quadratic$coeff.val)
  } else { # No grey zone detected, report kappa, AC2 and BP with linear and quadratic weights.
```

```
    agreement <- data.frame(Weights=c("Linear","Quadratic"),
                            Kappa=c(kappa.linear$coeff.val,kappa.quadratic$coeff.val),
                            AC2=c(AC2.linear$coeff.val,AC2.quadratic$coeff.val),
                            BP=c(BP.linear$coeff.val,BP.quadratic$coeff.val))
  }

  return(list(delta = delta, Delta = delta2, tau_Delta = tau_Delta,
              result = ifelse(delta2 > tau_Delta, "There is a grey zone.",
                              "There is not a grey zone."),
              agreement = agreement))
}
```

Examples

The following examples demonstrate the use of `detectGreyZone()` function.

Assessment of torture allegations data

```
data <- matrix(c(36,0,0,0,
                 7,57,11,0,
                 0,23,34,4,
                 0,1,19,10), nrow = 4, ncol = 4, byrow = TRUE)

detectGreyZone(data)
```

```
## Loading required package: irrCAC
```

```
## $delta
##          [,1]      [,2]      [,3]      [,4]
## [1,] 0.000000 -3.432727  0.000000  0.000000
## [2,] 3.432727  0.000000 -2.670128 -1.297449
## [3,] 0.000000  2.670128  0.000000 -4.058052
## [4,] 0.000000  1.297449  4.058052  0.000000
##
## $Delta
## [1] 4.058052
##
## $tau_Delta
## [1] 3.791366
##
## $result
## [1] "There is a grey zone."
##
## $agreement
##     Weights       AC2        BP
## 1 Quadratic 0.8834948 0.8653465
```

```
data2 <- matrix(c(9,32,8,1,0,
                  12,178,43,7,0,
                  1,42,61,25,0,
```

```r
                  0,1,10,17,2,
                  0,0,0,4,58), nrow = 5, ncol = 5, byrow = TRUE)

detectGreyZone(data2)
```

Assessment of PI-RADS v2.1 scores data

```
## $delta
##           [,1]       [,2]       [,3]      [,4]      [,5]
## [1,]  0.000000  4.6250711  3.5792459 1.533963  0.000000
## [2,] -4.625071  0.0000000  0.1663816 3.254026  0.000000
## [3,] -3.579246 -0.1663816  0.0000000 3.889305  0.000000
## [4,] -1.533963 -3.2540259 -3.8893049 0.000000 -1.252475
## [5,]  0.000000  0.0000000  0.0000000 1.252475  0.000000
##
## $Delta
## [1] 4.625071
##
## $tau_Delta
## [1] 4.537567
##
## $result
## [1] "There is a grey zone."
##
## $agreement
##     Weights       AC2        BP
## 1 Quadratic 0.9164416 0.8791585
```
